# The collaborative cross mouse for studying the effect of host genetic background on memory impairments due to obesity and diabetes

**DOI:** 10.1002/ame2.12488

**Published:** 2024-10-28

**Authors:** Avia Paz, Kareem Midlej, Osayd Zohud, Iqbal M. Lone, Fuad A. Iraqi

**Affiliations:** ^1^ Department of Clinical Microbiology and Immunology, Sackler Faculty of Medicine Tel Aviv University Tel Aviv Israel

**Keywords:** collaborative cross mouse, diabetes, host genetic background, memory impairments, obesity

## Abstract

**Background:**

Over the past few decades, a threefold increase in obesity and type 2 diabetes (T2D) has placed a heavy burden on the health‐care system and society. Previous studies have shown correlations between obesity, T2D, and neurodegenerative diseases, including dementia. It is imperative to further understand the relationship between obesity, T2D, and cognitive deficits.

**Methods:**

This investigation tested and evaluated the cognitive impact of obesity and T2D induced by high‐fat diet (HFD) and the effect of the host genetic background on the severity of cognitive decline caused by obesity and T2D in collaborative cross (CC) mice. The CC mice are a genetically diverse panel derived from eight inbred strains.

**Results:**

Our findings demonstrated significant variations in the recorded phenotypes across different CC lines compared to the reference mouse line, C57BL/6J. CC037 line exhibited a substantial increase in body weight on HFD, whereas line CC005 exhibited differing responses based on sex. Glucose tolerance tests revealed significant variations, with some lines like CC005 showing a marked increase in area under the curve (AUC) values on HFD. Organ weights, including brain, spleen, liver, and kidney, varied significantly among the lines and sexes in response to HFD. Behavioral tests using the Morris water maze indicated that cognitive performance was differentially affected by diet and genetic background.

**Conclusions:**

Our study establishes a foundation for future quantitative trait loci mapping using CC lines and identifying genes underlying the comorbidity of Alzheimer's disease (AD), caused by obesity and T2D. The genetic components may offer new tools for early prediction and prevention.

## Graphical abstract

Previous studies have shown correlations between obesity, type 2 diabetes (T2D), and neurodegenerative diseases, including dementia. A wide range of illnesses can result in dementia, including Alzheimer's disease (AD). The disorders that fall under the general category of “dementia” are caused by abnormal alterations in the brain. To counteract their significant influence on public health, it is imperative to gain an in‐depth understanding of the relationship between obesity, T2D, and cognitive deficits. We evaluate the cognitive impact of obesity and T2D induced by high‐fat diet (HFD) in collaborative cross (CC) mice. Our findings have demonstrated differences in the assessed phenotypes between the different CC lines and the C57BL/6J reference line. These findings highlight the role the host's genetic background plays in determining the degree of obesity and T2D development, as well as how it affects different organ weights and cognitive deficiencies that could worsen into AD when faced with a HFD challenge.

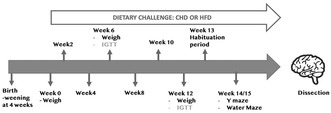

## 1 INTRODUCTION

The prevalence of obesity has alarmingly tripled in the past few decades. Studies carried out in the United States have demonstrated that this increase affects all racial and ethnic groups as well as men, women, and children.^1^ The health‐care system and society are heavily burdened by this notable increase in instances that negatively impact people's health.^2^ Although an individual's weight, height, and build are determined mainly by genetics, there is increasing agreement that environmental factors, rather than genetic makeup, are the primary cause of the obesity pandemic.^1^, ^3^ Modern Western culture, which encourages a sedentary and undernourished lifestyle known as an “obesogenic environment,” is blamed for this recent increase.^2^, ^4^ However, it is crucial not to disregard the genetic influence on obesity as genetic makeup interacts with environmental factors and may increase an individual's propensity.^5^, ^6^, ^7^ The obesogenic environment explains the increased prevalence seen over the past decades. Still, environmental factors alone do not rationalize why some people develop obesity whereas others maintain a moderate body weight when exposed to a similar environment.^6^ A complex interplay between genetics and the environment influences susceptibility to obesity.^3^, ^8^, ^9^, ^10^

The concern regarding obese individuals is that they are at a higher risk of developing obesity‐related comorbidities, such as metabolic syndrome and insulin resistance (IR).^11^, ^12^ These conditions are precursors to detrimental diseases such as type 2 diabetes (T2D), cardiovascular disease, hypertension, respiratory diseases, and certain types of cancers, which are more frequent among obese populations.^13^ Moreover, obesity has been found to accelerate the onset of several age‐related diseases, such as dementia, leading to earlier incidences of such diseases in response to obesity.^14^ Multiple diseases can cause dementia. It is an umbrella phrase used to characterize a range of symptoms in people with various conditions, including memory impairments (MI), which may lead to Alzheimer's disease (AD). Disorders categorized under the broad heading “dementia” are caused by abnormal changes in the brain.

T2D is one of the most associated comorbidities of obesity. Like obesity, T2D is influenced by the interaction between genetic elements and environmental factors. T2D is characterized by insulin sensitivity resulting from IR, decreased insulin production, and pancreatic β‐cell failure.^15^ In many cases, this development is impacted by obesity. T2D develops due to a decline in insulin secretion that cannot account for IR.^9^ This leads to decreased glucose transport into the liver, muscle, and fat cells. Statistical analysis reveals that 60%–90% of patients diagnosed with T2D are currently or have been obese. Obesity substantially influences IR, a condition that emerges in the early stages of T2D. A body mass index >35 kg/m^2^, deemed obese, increases the likelihood of developing T2D by 93‐fold among women and 42‐fold among men.^16^ Further research has revealed not only the connection between obesity and T2D but also a link between these diseases and cognitive impairments, such as MI and AD.^14^ Individuals diagnosed with T2D are at an increased risk of developing AD, primarily due to shared glucose metabolic abnormalities. Previous studies have identified an increased risk of developing dementia in correlation with a T2D diagnosis. Furthermore, T2D has been linked to the degradation of brain gray matter, increasing the risk of developing many brain diseases. Degradation of the hippocampus, an integral region in memory function, can lead to MI that may develop into AD later on.^17^ Further understanding the association between obesity, T2D, and cognitive impairments is vital in combating their considerable impact on public health. Identifying the mechanisms and the genetic components can aid in halting or preventing development.

Animal models are essential to comprehending the host's immune response.^18^, ^19^, ^20^ Researchers must determine whether sufficient data exist to support using an animal model for research, whether ethical concerns are addressed, and whether the information gathered from animal studies will significantly advance scientific understanding before they select an animal model.^18^, ^21^ To induce the disease state, the host, ranging from fish to mice, must be artificially manipulated.^22^, ^23^

To explore the role of genetic background in MIs due to obesity and diabetes, the Collaborative Cross (CC) mouse genetic reference population must be implemented, which provides a powerful murine model for studying and analyzing the genetics of complex diseases, such as diet‐induced obesity and T2D, as shown in several studies conducted by our team and collaborators.^24^ The CC mouse population represents a promising resource in genetics, offering a platform for investigating the complex interaction between genetic variation and phenotypic diversity.^25^, ^26^

The CC population was developed by community efforts of the Complex Trait Consortium (www.complextrait.org). This unique genetic resource eventually comprises a set of recombenant inbred lines (RIL that will be produced by full reciprocal eight‐way mating of eight divergent strains of mice: A/J, C57BL/6J, 129S1/SvImJ, NOD/LtJ, NZO/HiLtJ, CAST/Ei, PWK/PhJ, and WSB/EiJ. Controlled randomization and minimization of selection during the breeding process recombined the natural genetic variation presented in these inbred strains. The result is a unique collection of RIL exhibiting a large phenotypic and genetic diversity and bringing the tremendous genetic variation potential of the mouse inbred lines to phenotypic expression.^27^ One of the primary advantages of the CC mouse population lies in its ability to capture the full breadth of genetic variation present in outbred mouse populations.^28^, ^29^ Unlike traditional laboratory mouse strains characterized by limited genetic diversity and fixed allelic compositions, CC lines exhibit extensive allelic diversity and genetic heterogeneity.^30^ This genetic complexity mirrors the diversity in human populations and provides a rich substrate for dissecting the genetic framework of complex traits.^31^

In previous studies, males and females of the CC mouse line populations varied in their response to T2D development induced by a high‐fat diet (HFD).^32^ Thus, it is confirmed that the effects of sex and diet are evident among the different lines. However, some lines had a more significant sex difference than others, and different CC lines exhibited different susceptibilities.^33^ Limited genetic diversity of current animal models often used in genetic mapping of complex trait diseases, including T2D, obesity, and AD, presents a challenge for researchers to form a comprehensive network that incorporates genetic interactions, epigenetics, environmental factors, microbiota, and other phenotypes. The CC represents a genetically high‐diversity resource that analyzes complex traits, including chronic and infectious diseases.^34^

In our previous studies focusing on mapping novel quantitative trait loci (QTL) associated with glucose tolerance, we identified several key genes, including *Adrb3* and *PGK1*, which play crucial roles in determining the degree of obesity and T2D development.^32^ These findings highlight the genetic factors influencing glucose metabolism and suggest potential therapeutic targets for managing T2D and related metabolic disorders.^32^ Additionally, our prior research identified important genes such as *APOL6* and *APOL8*, which are involved in adipocyte and neuronal differentiation, respectively, and influence the development of obesity and T2D.^32^ Furthermore, candidate genes like *Trap1* and *Rrn3* were found to be associated with body weight regulation, providing insights into the genetic framework underlying these metabolic conditions.^35^ These previous findings focused on the impact of genetic markers on body weight changes in response to a HFD, shedding light on the genetic factors contributing to obesity and T2D progression.^35^ Collectively, the identification of these genetic markers is essential for understanding the complex nature of T2D and the genetic basis of its associated conditions, such as metabolic disorders, providing valuable insights for future research and personalized medicine approaches.

This project implemented several experiments to assess the HFD‐induced T2D effect on cognition. This research project aims to understand further the correlation among obesity, T2D, and cognitive impairment and the effect of host genetic background on defining the severity of this comorbidity. Experiments have been conducted to check glucose tolerance, cognition, and learning.^36^ Understanding the underlying genes and mechanisms of obesity and its associated health consequences may help mitigate the consequences and potential risks of age‐related diseases, such as AD.^33^, ^37^ These results establish and provide a basis for gene mapping for QTLs and subsequently identify candidate genes responsible for multimorbidity of obesity, T2D, and AD.

## 2 MATERIALS AND METHODS

### 2.1 Ethical standards

All animals were treated under the standards of care and use of laboratory animals. Mice were housed in an environment of a 12‐h light–dark cycle and provided with food and water ad libitum. The experiments were approved by the Institutional Animal Care and Use Committee (IACUC) of Tel Aviv University (TAU), Israel (approval number: TAU‐MD‐IL‐2205‐160‐5).

**FIGURE 1:**
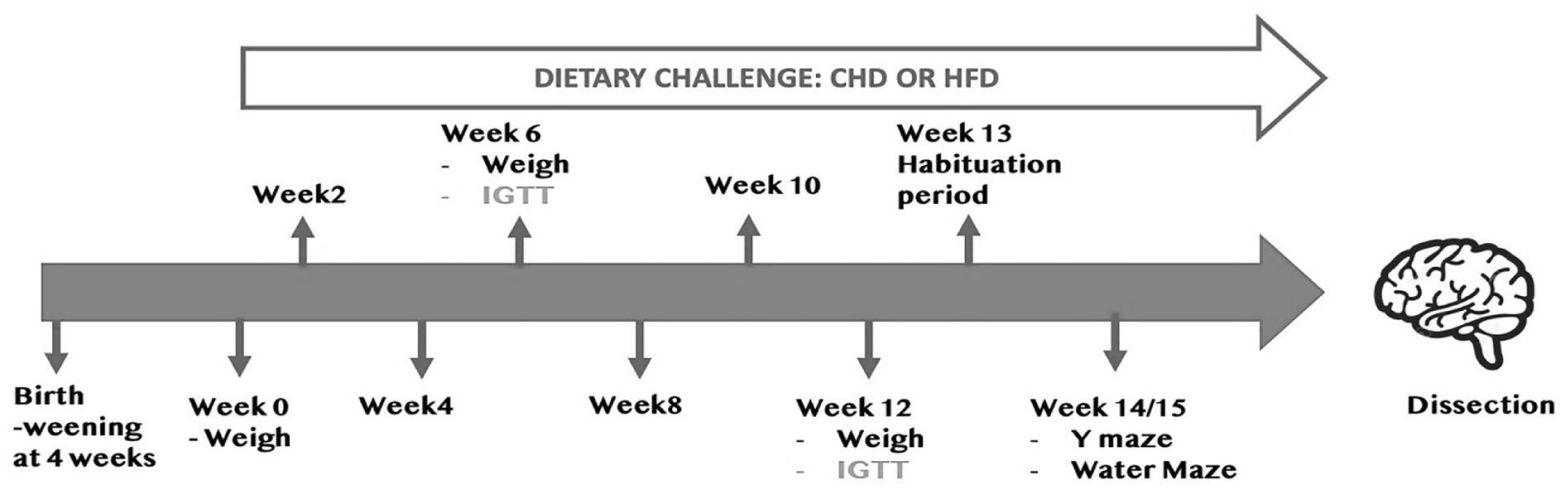
Experimental design. Mice were weaned 4 weeks postbirth; at 8 weeks, the experiment begins and is referred to as week 0 of the experiment. Biweekly weighing and IPGTTs (intraperitoneal glucose tolerance tests) were conducted on weeks 6 and 12. After the 12‐week diet challenge, mice were moved to the Core Behavioral Unit for a 1‐week habituation period. Y maze test was conducted, followed by the Morris water maze (MWM) test. The brain, kidney, spleen, and liver were dissected and weighed at the end.

The experiments spanned 14 weeks. Mice were weaned 4 weeks postbirth, and the experiments started in the eighth week. One half the cohorts were fed a HFD, whereas the other half, used as a control, were fed a chow diet (CHD). The mice were weighed biweekly, and an intraperitoneal glucose tolerance test (IPGTT) was conducted at two time points, weeks 6 and 12 of the experiment. Y maze and Morris water maze (MWM) were performed after a 1‐week habitation period after week 12 of the experiment. Mice were killed, and the brain, liver, kidney, and spleen were collected and weighed. This experimental process is shown in Figure 1.

We assessed multiple groups of CC mice to address different experimental conditions and the diverse genetic backgrounds of the CC lines.^20^ This approach confirms that the host genetic background is crucial in determining the studied phenotypes.^20^ Additionally, it enables us to define and estimate heritability accurately. By using a wide array of CC lines, we can better understand the genetic basis of traits related to obesity, T2D, and MIs.^26^

### 2.2 Dietary challenge

The experimental group (aged 8 weeks) was fed a high‐fat Western diet (HFD) (TD. 88 137, 42.0% kilocalories from fat, 15.3% from protein, and 42.7% from carbohydrates, mainly sucrose [Teklad Global, Harlan Inc.]) for the entire experimental procedure. The control group was fed a standard CHD, Altromin 1324 IRR diet (Altromin Spezialfutter GmbH & Co, Germany), consisting of 11% kilocalories from fat, 24% from protein, and 65% from carbohydrates.

### 2.3 Physical body measurements

Throughout the experimental period, mice had free access to water and diet. Body weight was recorded biweekly.

### 2.4 IPGTT and area under the curve calculation

The aim of this test was to identify abnormalities in glucose metabolism that may be associated with diabetes or prediabetic disorders, as described in our earlier articles.^23^

### 2.5 Y maze

The test was performed using a symmetrical white Plexiglas Y maze with three arms defined as start, familiar, and novel after exploring the familiar arm and a break. The mice were reintroduced into the maze with two arms, familiar and novel. The maze was cleaned between sessions. Familiar and novel arms were changed randomly. Figure S1 shows the experimental procedure.

### 2.6 Morris water maze

The MWM test was conducted in a pool with a transparent platform placed at one end in the center of the axis. The mice were trained over 5 days, and the probe test was on day 6. Each day, mice were placed thrice in the maze in three different quadrants for 3 min, each run spanning 1 min. If the mice did not find the platform, they were directed to it by the experimenter and were kept there for 20 s before being placed again in the maze. EthoVision software tracked the movements of the mice (in MWM and Y maze) and provided data on the time spent and frequency.

On day 6, the platform was removed. Mice were placed in the region opposite the platform once for 1 min. The software recorded the areas in which they were found. Figure S2 shows the experimental procedure.

### 2.7 Mouse dissection

The brain, kidney, liver, and spleen tissues were dissected and weighed at the end of the experiment. The number of mice (*n*) that were involved in the experiments (not the behavioral experiments) and the dietary challenge maintained on CHD/HFD are presented in Table 1. Table 2 presents the number of mice (*n*) involved in the experiments (including behavioral experiments) and the dietary challenge maintained on CHD/HFD.

**TABLE 1:** The number of mice (*n*) that underwent the experimental protocol (not the behavioral experiments) and the dietary challenge maintained on CHD/HFD.

CC line	HFD		CHD		Total
	Female	Male	Female	Male	
CC037	9	3	22	11	45
CC019	6	10	8	7	31
CC010	7	7	3	7	24
CC005	11	25	20	22	78
C57BL/6	4	3	12	11	30
					208

Abbreviations: CHD, chow diet; HFD, high‐fat diet.

**TABLE 2:** The number of mice (*n*) that underwent the experimental protocol (including behavioral experiments) and the dietary challenge maintained on CHD/HFD.

CC line	HFD diet		CHD diet		Total
	Female	Male	Female	Male	
CC037	7	3	18	11	39
CC019	2	9	3	7	21
CC010	4	5	3	7	19
CC005	11	25	16	20	72
C57BL/6	3	3	7	8	21
					172

Abbreviations: CHD, chow diet; HFD, high‐fat diet.

### 2.8 Heritability

Heritability measures the fraction of phenotype variability attributed to genetic variation. Here, we used the analysis of variance (ANOVA) results to calculate the broad‐sense heritability using the following formula:

$$H2=\mathrm{Vg}/\left(\mathrm{Vg}+\mathrm{Ve}\right)$$

where *H*2 is the heritability, *Vg* is the genetic variance between the CC lines, and *Ve* is the environmental variance.^17^ Data analysis was performed using R language software. Wilcoxon test is a nonparametric test used to compare two independent samples, whereas Kruskal–Wallis test is a nonparametric test for multiple comparisons. Tukey's honestly significant difference post hoc analysis was performed to assess the significant differences between challenges and conditions among the assessed traits. Spearman's correlation was calculated as the correlation coefficient between the different measured traits.

## 3 RESULTS

Our study aimed to investigate the impact of host genetic background on the development of obesity, T2D, cognitive deficits, and potential progression to AD in response to a HFD challenge using CC mice. Our results revealed significant variations in phenotypic responses among different CC mouse lines, highlighting the crucial role of genetic diversity in determining susceptibility to diet‐induced metabolic and cognitive disorders. Specifically, we observed diverse body weight, glucose tolerance, organ weight, and cognitive function responses among the CC mouse lines after a HFD. These findings provide valuable insights into the complex interplay between genetic factors and environmental stimuli in the pathogenesis of metabolic and cognitive disorders. Moreover, our results have implications for understanding the genetic basis of disease susceptibility and developing personalized therapeutic interventions targeting obesity, T2D, and cognitive impairments.

### 3.1 Variation in body weight

**FIGURE 2:**
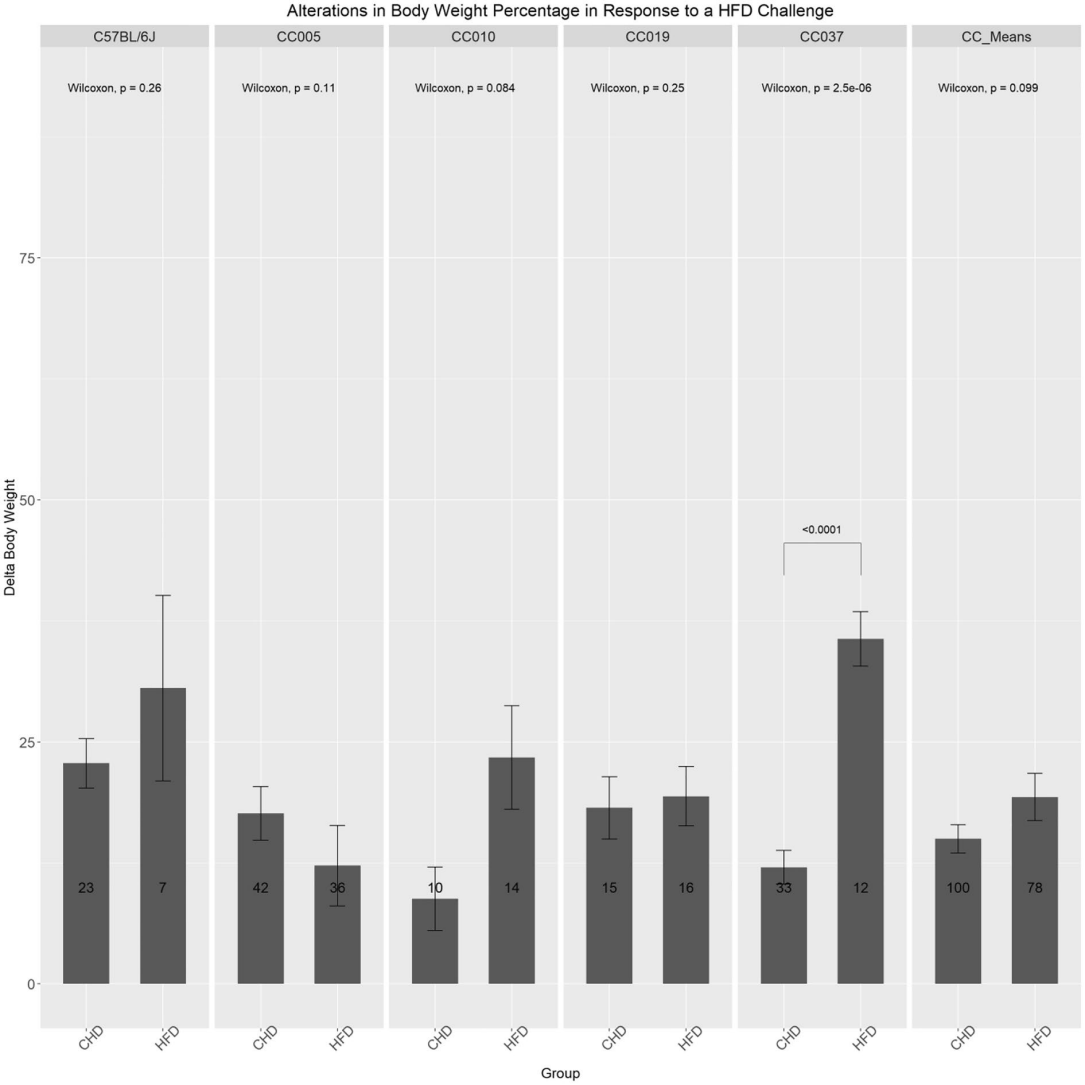
Alterations in percentage delta body weight response to the HFD (high‐fat diet) challenge among the CC (collaborative cross) lines and C57BL/6J controls. The Figure  shows the body weight changes (g) after 12 weeks of diet challenge CHD (chow diet) versus HFD. The *x*‐axis represents the HFD of four different CC lines, the means of the overall population, and C57BL/6J. The *y*‐axis represents ∆BW(g). The statistical significance of *p*‐values is indicated if found significant.

Figure 2 shows that C57BL/6J exhibited an increase in percentage delta body weight in response to a HFD. The increases among CC010 and a highly significant (*p* < 0.001) increase among CC037 were also observed when mice of these lines were maintained on a HFD. Line CC05 revealed a decrease in percentage delta body weight in response to a HFD, whereas CC019 exhibited a slight increase due to the HFD. Furthermore, Figure 2 shows that the CC mean exhibited an increase in percentage delta body weight among mice maintained on a HFD compared to mice maintained on a CHD.

Figure S3A shows five mouse lines presenting alternative responses to the dietary challenge. Sex effect is evident as male and female mice of each line also differ in their response to the dietary challenge. C57BL/6J female mice revealed a decrease in percentage delta body weight in response to the HFD, whereas male mice of the same line exhibited an increase in response to the HFD. Line CC005 exhibited an increase in the HFD among female mice; however, male mice of this line exhibited a decrease in delta body weight in response to the HFD. Line CC010 mice exhibited an increase in body weight in response to a HFD in both female and male mice. Line CC019 female mice exhibited a decrease in body weight in response to the HFD, whereas male mice of this line exhibited a minor increase in body weight in response to the HFD. Line CC037 female mice exhibited a significant increase when maintained on a HFD compared to CHD controls. Similarly, male mice of this line exhibited a significant increase in body weight on a HFD. Moreover, Figure S3B shows the CC mean when the sex effect is considered; female mice exhibited an increase in body weight on a HFD, whereas male mice exhibited a slight decrease in delta body weight.

### 3.2 Variation in glucose tolerance

**FIGURE 3:**
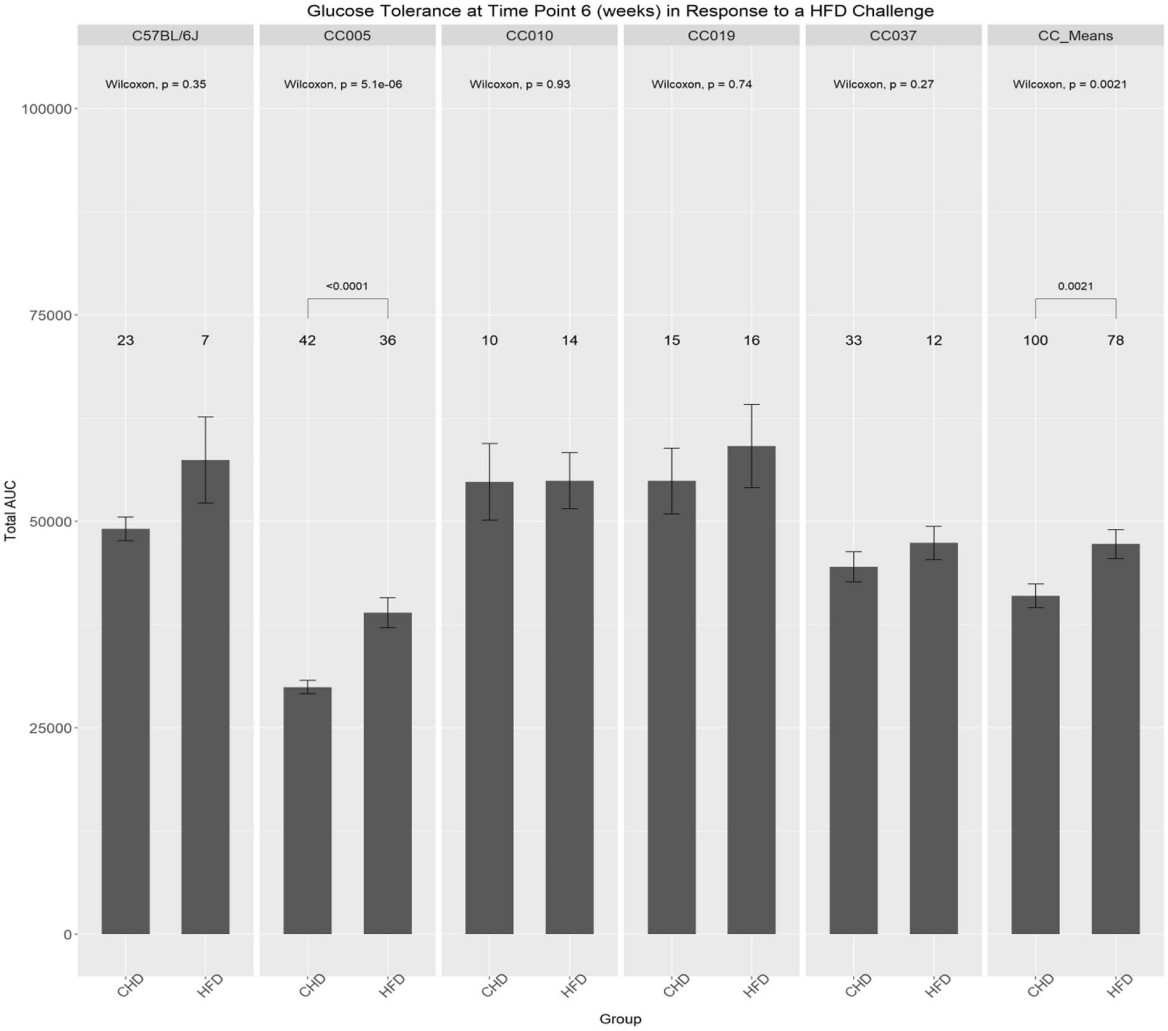
Influence of the dietary challenge on glucose tolerance at 6 weeks among the CC (collaborative cross) lines and C57BL/6J controls. The total area under the curve (AUC_0–180_) of glucose clearance (min mg/dL) of intraperitoneal glucose tolerance test (IPGTT) at week 6 of four different CC lines, the means of the overall population, and C57BL/6J after maintenance on either HFD (high‐fat diet) or CHD (chow diet). The *x*‐axis represents CC lines and the means, whereas the *y*‐axis represents the AUC. Significant *p*‐values are indicated.

Figure 3 shows that all lines tested possess higher area under the curve (AUC) values among mice maintained on a HFD than their CHD counterparts. Notably, line CC005 exhibited a highly significant (*p* < 0.001) increase when mice of this line on a HFD was compared with those in the CHD group. Furthermore, the CC_Means of all texted mice   exhibited a significant increase in AUC value among mice maintained on a HFD compared to those maintained on a CHD.

Figure S4A shows the altered responses in AUC values at six time points among the lines, and the sex effect is present. C57BL/6 female mice exhibited a slight increase in AUC values among males of this line. The overall AUC values of the total population were increased in mice on a HFD compared to a CHD; however, the difference was insignificant. Nonetheless, different CC lines exhibited variations in the AUC of the mice maintained on a HFD and CHD. For instance, line CC005 exhibited an increase in AUC values on a HFD in both female and male mice, although the increase in male mice was significant. Similarly, lines CC010 and CC037 exhibited an increase in AUC values on a HFD; however, the difference was insignificant. Line CC019 exhibited a decrease in AUC values in female and male mice in response to the HFD challenge. Moreover, Figure S4B shows that the CC mean exhibited an increase in the AUC values of females and males maintained on a HFD compared to CHD counterparts at week 6.

**FIGURE 4:**
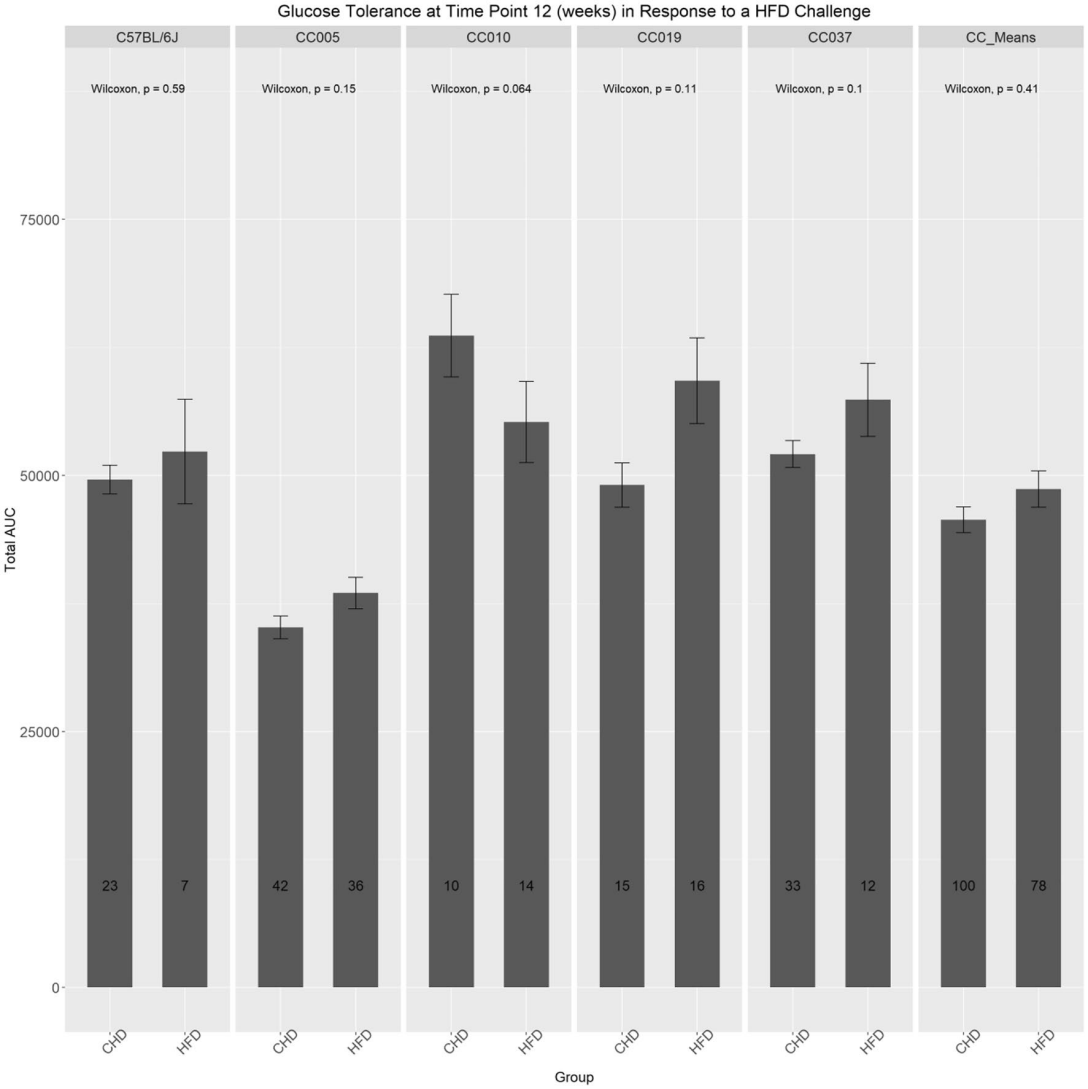
Influence of the dietary challenge on glucose tolerance at 12 weeks among the CC (collaborative cross) lines and C57BL/6J controls. The total area under the curve (AUC_0–180_) of glucose clearance (min mg/dL) of intraperitoneal glucose tolerance test (IPGTT) at week 12 of four different CC lines and C57BL/6 controls after maintenance on either HFD (high‐fat diet) or CHD (chow diet). The *x*‐axis represents CC lines and the mean, whereas the *y*‐axis represents the AUC. Significant *p*‐values are indicated. AUC_0–180_ of glucose clearance (min mg/dL) of IPGTT at week 12 of the CC mean after maintenance on either HFD or CHD. The *x*‐axis represents CC lines and the mean, whereas the *y*‐axis represents the AUC. Significant *p*‐values are indicated.

Figure 4 shows the altered responses among the different lines tested. Lines C57BL/6J, CC010, CC019, and CC037 exhibited an increase in AUC values on a HFD compared to their CHD counterparts. Conversely, CC010 exhibited a decrease in AUC in response to the HFD. Furthermore, Figure 4 shows an increase in AUC values in response to a HFD. The results in Figure S5A show that different lines responded differently to the HFD with an add‐on sex effect. Lines C57BL/6, CC005, CC019, and CC037 exhibited an increase in AUC values in response to a HFD in both male and female mice. Conversely, CC010 exhibited decreased AUC values in response to the HFD in female and male mice of this line. Furthermore, Figure S5B shows that overall, the CC mean exhibited a slight increase, with almost no change in the AUC values in response to the HFD challenge.

### 3.3 Variation in organ weight

**FIGURE 5:**
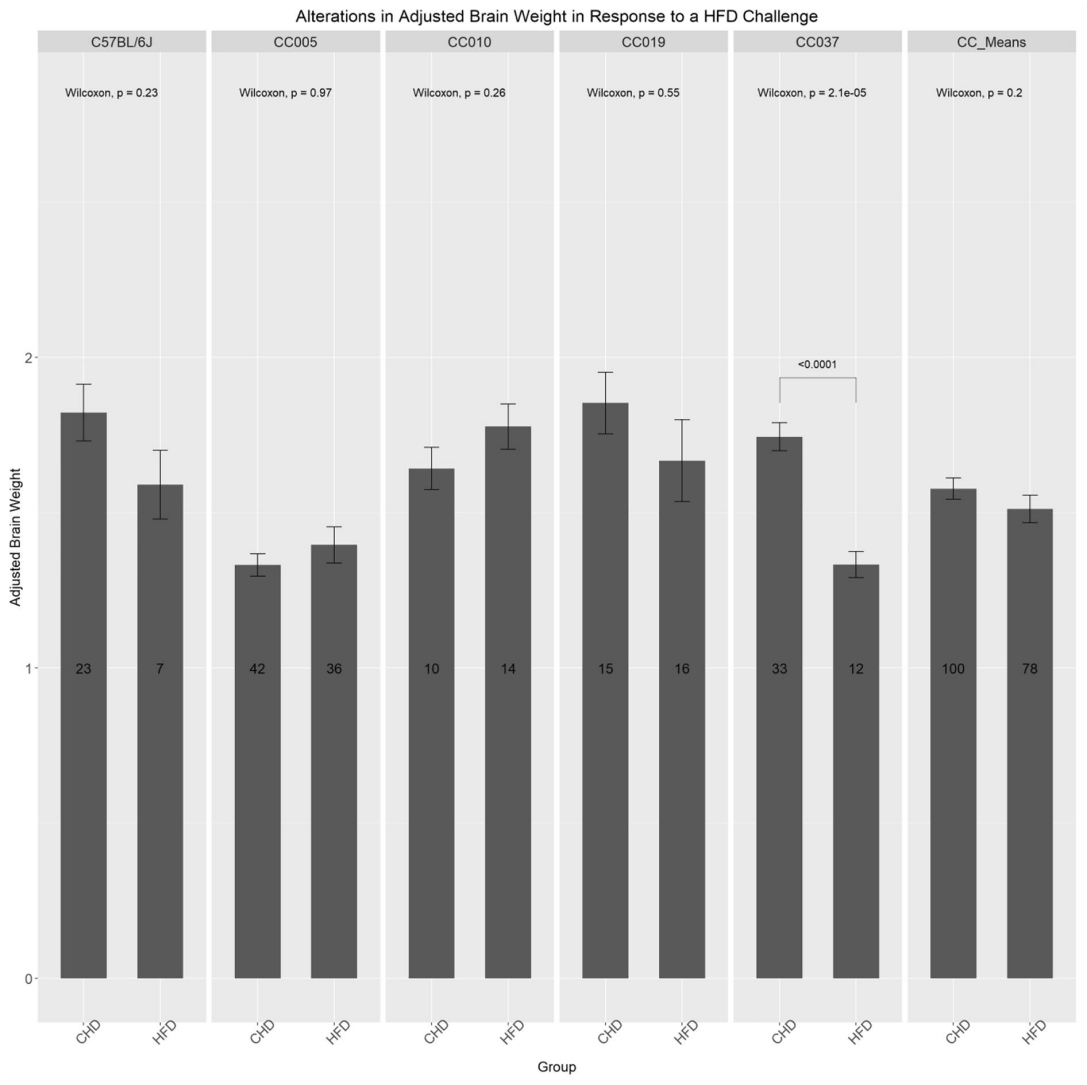
Influence of the dietary challenge on adjusted brain weight among the CC (collaborative cross) lines and C57BL/6J controls. The brain weight collected at the end of the experiment and adjusted to the final body weight (adjusted brain weight) of CC line mice and C57BL/6 after maintenance on either HFD (high‐fat diet) or CHD (chow diet). The *x*‐axis represents CC lines and C57BL/6 controls; the *y*‐axis represents the adjusted brain weight. Significant *p*‐values are indicated; Kruskal–Wallis + Dunn's test multiple comparisons were conducted. The brain weight collected at the end of the experiment and adjusted to the final body weight (adjusted brain weight) of the CC mean after maintenance on either HFD or CHD. The *x*‐axis represents CC lines and C57BL/6 controls; the *y*‐axis represents the adjusted brain weight. Significant *p*‐values are indicated; Kruskal–Wallis + Dunn's test multiple comparisons were conducted.

Figure 5 shows the adjusted brain weight to the total body weight of the mouse at the dissection time, which was affected differently among the different lines in response to a HFD. Lines C57BL/6J, CC019, and CC037 exhibit a decrease in adjusted brain weight in response to the diet with line CC037, which was found to be highly significant (*p* < 0.001). Lines CC005 and CC010 indicate a slight increase in the adjusted brain weight in response to the HFD. Moreover, Figure 5 shows that the CC mean indicates, overall, a decrease in adjusted brain weight in response to the HFD compared to CHD‐maintained mice. Figure S6A,B shows that the adjusted brain weight was influenced differently among the lines and between different sexes. Lines C57BL/6J and CC037 exhibited a decreased adjusted brain weight in response to a HFD in both sexes. In the case of spleen, shown in Figure S7A,B, the altered weights were recorded among the different lines, revealing a genetic impact on the dietary challenge. Line CC005 exhibited a significant increase in spleen weight among males maintained on a HFD compared to CHD counterparts. The female mice of this line exhibited a slight decrease in response to the HFD. Line CC010 exhibited an increase among female and male mice maintained on a HFD. Line CC019 exhibited an increase among both female and male mice maintained on a HFD compared to CHD counterparts.

Liver results, in Figure S8A,B, reveal that despite a constant environment, alterations in liver weight are evident. C57BL/6 exhibited a decrease in liver weight among female mice, whereas an increase was observed among males of this line. A highly significant (*p* = 0.001) difference was observed between the liver weight of female mice of this line maintained on a HFD compared to male mice of this line on a HFD. Lines CC005 and CC019 exhibited an increase in liver weight in both male and female mice in response to a HFD compared to CHD counterparts.

Figure S9A,B shows that the kidney results reveal various responses in kidney weight elicited among the lines in response to the HFD. C57BL/6 female mice exhibited a significant decrease in kidney weight when maintained on a HFD compared to CHD counterparts. Male mice of this line exhibited increased kidney weight in response to the HFD. Similarly, line CC005 exhibited a decrease in female mice and an increase in male mice as a response to the HFD.

### 3.4 Estimating the heritability of the assessed phenotypes

The heritability of the assessed phenotypes was estimated, and results are presented in Tables 3 and 4. Table 3 presents the heritability of female mice maintained on a CHD and HFD. This study aimed to assess if the phenotypic variance observed has a genetic basis due to the dietary challenge. Table 3 summarizes the calculated heritability (*H*2) values. One‐way ANOVA was used to calculate the heritability of female‐ and genotype‐specific characteristics of several parameters: body weight initial; body weight final; delta body weight; AUC 6 and AUC 12; and adjusted brain, liver, and spleen weight. Similarly, Table 4 presents the heritability results of male mice maintained on a CHD and HFD. Figure S10A,B shows that the HFD impacts the preference index, and a line effect is present. All the lines exhibited a higher preference index on a HFD than a CHD counterpart. However, the ratios are different. Figure S11A,B shows that the HFD impacts the preference index, and a line and sex effect is present. Line CC037 exhibited a significantly higher preference index among male mice on a HFD compared to CHD counterparts.

**TABLE 3:** Heritability—female mice maintained on CHD and HFD: Summary of the calculated heritability (*H*2) values.

	Trait	*H*2	CVg	ANOVA Sig
Female CHD	Body weight initial	0.59	0.12	0.000
	Body weight final	0.73	0.12	0.000
	Delta body weight			0.344
	Total AUC week 6	0.53	0.26	0.000
	Total AUC week 12	0.73	0.24	0.000
	Adjusted brain weight	0.48	0.14	0.000
	Adjusted liver weight	0.22	0.10	0.008
	Adjusted spleen weight			0.312
	Adjusted kidney weight			0.283
Female HFD	Body weight initial	0.43	0.11	0.001
	Body weight final	0.47	0.14	0.001
	Delta body weight	0.39	0.49	0.003
	Total AUC week 6	0.75	0.24	0.000
	Total AUC week 12	0.49	0.20	0.000
	Adjusted brain weight	0.65	0.19	0.000
	Adjusted liver weight	0.42	0.22	0.002
	Adjusted spleen weight	0.3	0.43	0.014
	Adjusted kidney weight			

*Note*: One‐way ANOVA was used to calculate the heritability of female‐ and genotype‐specific characteristics of several parameters: body weight initial; body weight final; delta body weight; AUC 6; AUC 12; and adjusted brain, liver, and spleen weight.

Abbreviations: ANOVA, analysis of variance; AUC, area under the curve; CHD, chow diet; CVg, genetic covariance; HFD, high‐fat diet; Sig, Significant.

**TABLE 4:** Heritability—male mice maintained on CHD and HFD: Summary of the calculated heritability (*H*2) values.

	Trait	*H* ^2^	CVg	ANOVA Sig
Male CHD	Body weight initial	0.55	0.11	0.000
	Body weight final	0.55	0.13	0.000
	Delta body weight			0.133
	Total AUC week 6	0.70	0.32	0.000
	Total AUC week 12	0.72	0.27	0.000
	Adjusted brain weight	0.53	0.16	0.000
	Adjusted liver weight	0.53	0.12	0.000
	Adjusted spleen weight			0.316
	Adjusted kidney weight	0.39	0.12	0.000
Male HFD	Body weight initial	0.64	0.14	0.000
	Body weight final	0.31	0.11	0.003
	Delta body weight			0.311
	Total AUC week 6	0.36	0.22	0.001
	Total AUC week 12	0.55	0.28	0.000
	Adjusted brain weight			0.082
	Adjusted liver weight			0.544
	Adjusted spleen weight			0.189
	Adjusted kidney weight	0.15	0.13	0.049

*Note*: One‐way ANOVA was used to calculate the heritability of male‐ and genotype‐specific characteristics of several parameters: body weight initial; body weight final; delta body weight; AUC 6; AUC 12; and adjusted brain, liver, and spleen weight. Maintained on either HFD (42% fat) challenge or CHD (18).

Abbreviations: ANOVA, analysis of variance; AUC, area under the curve; CHD, chow diet; CVg, genetic covariance; HFD, high‐fat diet; Sig, Significant.

### 3.5 Behavioral tests

#### 3.5.1 MWM over the span of 6 days

The following results present the 6‐day MWM test of each line tested after maintenance on either a HFD or CHD.

#### 3.5.2 MWM days 1–6 of line CC0037

**FIGURE 6:**
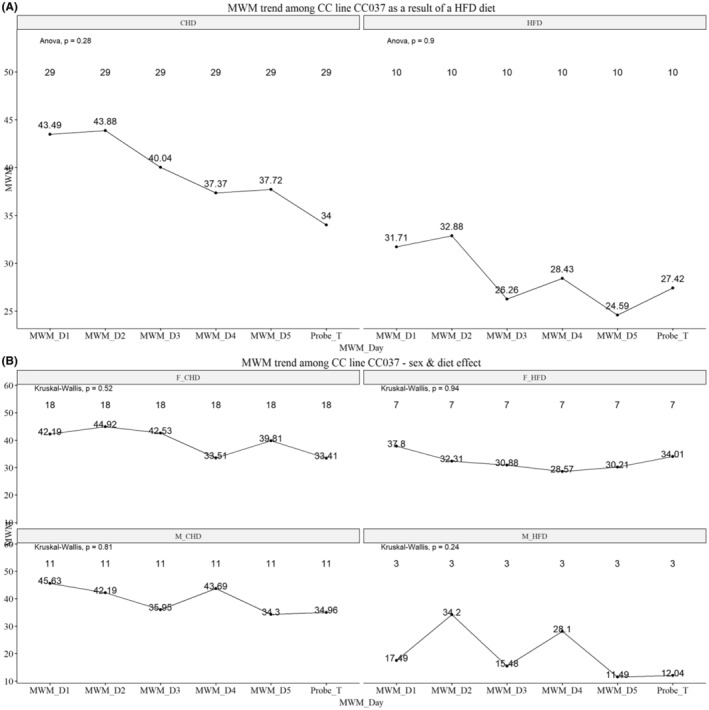
Morris water maze (MWM) days 1–6 of CC (Collaborative Cross) line CC0037. (A) The dietary challenge impacts the mice of line CC037 as the execution of the maze is different when the diets are compared. The graphs represent the 6‐day MWM of each line tested after maintenance on either HFD (high‐fat diet) or CHD (chow diet). Panel (A) represents the diet effect; (B) the sex and diet effect for each line. The *x*‐axis represents the days (1–5) and the probe test on day 6; the *y*‐axis represents time in seconds. Significant *p*‐values are indicated.

Figure 6A shows that the dietary challenge impacts the CC037 line mice, as the execution of the maze test is different when compared to the diets. Mice maintained on a CHD learn the maze, and an improvement is evident as days progress. Mice maintained on a HFD exhibit a less‐steady improvement, but overall, the performance is better on a HFD. Moreover, Figure 6B shows the variation in response to the diet between female and male mice when sex and diet are considered. The diet appears to impact male mice of the CC037 line more than females. Males of this line perform the maze test better when on a HFD.

#### 3.5.3 MWM days 1–6 of line CC005

The results in Figure S12A show that the dietary challenge does not influence the overall performance of the CC005 line, as the progress in learning the maze is similar. Figure S12B shows that there is variation in response to the diet between female and male mice when sex and diet are considered together. Yet the overall progression is similar among male and female mice.

#### 3.5.4 MWM days 1–6 of line CC019

Figure S13A shows that the dietary challenge impacts the line CC019 mice as the performance of the maze test is different when the diets are compared. Mice maintained on a CHD exhibited an inconsistent learning curve and a regression in performance. Mice maintained on a HFD exhibited an inconsistent learning curve, which improved over the days. Overall, the performance is better on a HFD; Figure S13B shows that there is variation in response to diet and between female and male mice when sex and diet are considered together. An improved performance is evident among female mice maintained on a HFD, with improved performance also in males in response to the HFD.

#### 3.5.5 MWM days 1–6 of line CC010

Figure S14A shows that the dietary challenge impacts the line CC010 mice as the performance of the maze test differs from the diets. The learning curve of mice maintained on a CHD and HFD could be more steady. Both reveal a regression in performance. Figure S14B shows that when sex and diet are considered together, there is variation in response to the diet between female and male mice. The diet appears to impact the performance of females on a HFD as the regression is negative; however, an improved performance is observed in males on a HFD compared to CHD counterparts.

#### 3.5.6 MWM days 1–6 of line C57BL/6J

Figure S15A shows that the dietary challenge impacts the line C57BL/6J mice as the performance of the maze test is different compared to the diets. Mice maintained on a CHD exhibit a steady learning curve, whereas HFD‐maintained mice exhibit a slight regression. Ultimately, this line performs the maze test well. Figure S15B shows that when sex and diet are considered together, there is variation in response to the diet and between female and male mice. However, all females and males of this line perform the maze test well, and an improvement is observed as days progress on both the CHD and HFD.

Figure S16A shows that among all the four CC lines tested, the HFD improved the execution, reaching the platform region. Mice maintained on a HFD reached the probe region faster than those maintained on a CHD. Conversely, C57BL/6 on a CHD performed the test slightly faster. Furthermore, Figure S16B shows that, overall, the CC mean of mice on a HFD exhibited improved performance in reaching the platform region compared to CHD counterparts.

#### 3.5.7 Heat maps of the CC lines

**FIGURE 7:**
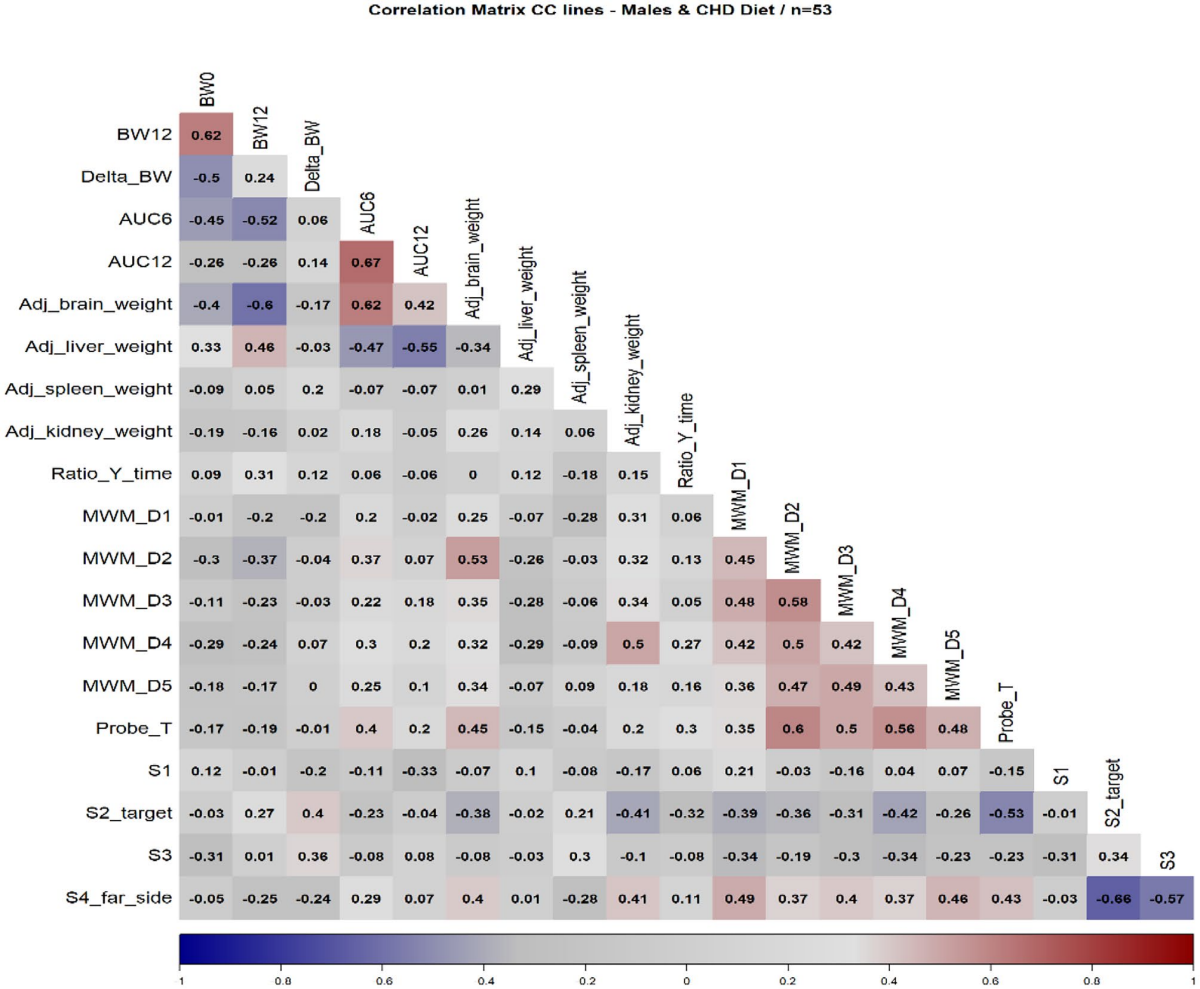
Heat map and Spearman's correlation for CC (Collaborative Cross) mean of select traits of the CC population after being maintained for 14 weeks on either a HFD (high‐fat diet) or CHD (chow diet). The figure represents male mice maintained on CHD. The *r* values ranged between minimum (−1) and maximum (1).

The correlation results in Figure 7 show a heat map and Spearman's correlation for the CC mean of select traits of the CC population after being maintained for 14 weeks on either a HFD or CHD. Figure 7 shows male mice maintained on a CHD (Figure S17), male mice maintained on a HFD (Figure S18), female mice maintained on a CHD, and female mice maintained on a HFD (Figure S19). The *r* values ranged between minimum (−1) and maximum (1).

These correlations reveal altered responses among traits assessed in response to the HFD and sex effect. A negative correlation is observed among adjusted liver weight and AUC 6 and AUC 12 among male CC mice on a CHD, whereas the correlation neutralizes in response to a HFD. Moreover, a positive correlation is observed among male mice on a CHD between adjusted brain weight and AUC 6. Similarly, this correlation neutralizes on a HFD. The adjusted kidney weight and BW12 are neutral on a CHD among male mice; however, a negative correlation is observed on a HFD. Among female mice the adjusted kidney weight also changes from a neutral correlation to BW12 on a CHD to a negative correlation on a HFD. Similarly, adjusted liver weight and delta BW negatively correlate with a HFD, whereas the correlation is neutral on a CHD.

Figure S20 shows the heat map and Spearman's correlation for select traits of the C57Bl/6J line after being maintained for 14 weeks on either a HFD (Figure S20B) or CHD (Figure S20A). The *r* values ranged between minimum (−1) and maximum (1). These correlation matrices reveal the influence of the HFD challenge on C57Bl/6J. A weak positive correlation between AUC 12 and BW12 is observed when maintained on a CHD. Conversely, C57Bl/6J mice maintained on a HFD reveal a strong positive correlation.

## 4 DISCUSSION

Our study aimed to investigate the role of genetic background in mediating the phenotypic response to a HFD challenge using CC mice. The CC mouse model has been shown in previous studies to be a potent tool for research in complicated diseases.^34^, ^38^, ^39^, ^40^ A thorough examination of complex traits within the framework of systems genetics can be achieved by putting this approach into practice. The CC mouse model demonstrated improved power in exploring the interaction between genetics and disease development compared to previously used approaches.^41^, ^42^ The findings presented in this study shed light on the complex interplay between genetic factors and environmental stimuli in the development of obesity, T2D, cognitive deficits, and potential progression to AD.

Compared to other recombinant mouse inbred strains, the CC mice exhibited a notably higher level of recombination in their genetic makeup. The population's extensive genetic diversity, with over 36 million single‐nucleotide polymorphisms segregating, represented a key feature of the CC mouse model, enhancing its efficacy in gene mapping abilities.^38^ The reason for assessing multiple groups of CC mice is to address the different experimental conditions and genetic backgrounds. This comprehensive approach ensures that we can confirm the significant impact of the host genetic background on the studied phenotypes. By examining diverse lines with varying genetic compositions, we can better define and estimate heritability. This methodology allows for a more robust analysis of the genetic factors influencing obesity, T2D, and related MIs, providing valuable insights into the complex interplay between genetics and these conditions.

One of the key findings of our study is the significant variability observed in the phenotypic response to the HFD challenge among different CC mouse lines. This variability underscores the importance of genetic background in shaping individual susceptibility to diet‐induced metabolic and cognitive disorders. Our results highlight the role of genetic diversity represented by the CC mouse panel in elucidating the complex genetic framework underlying these disorders.

The global increase in obesity cases is attributed primarily to an unhealthy diet and lifestyle.^1^, ^2^, ^43^ A diet high in fat and low in nutrients results in weight gain among most individuals.^44^ Our results reveal that overall, among the CC lines, without taking sex into account, a HFD increases the percentage change in body weight. Although an obesogenic environment promotes weight gain, not all individuals exposed to such an environment become obese; some remain lean. The variation in a consistent climate indicates that some are resistant to weight gain, whereas others are susceptible; this phenomenon is reflected in our results.^45^ When line effect is considered, C57BL/6J, CC010, and CC037 lines exhibited an increase in response to the HFD, whereas line CC019 exhibited a minor increase, with almost no change in response to the HFD. Conversely, line CC005 exhibited a decrease in weight because of maintenance on a HFD compared to CHD counterparts. Moreover, when the sex effect is considered, variations between lines are evident. C57BL/6J female and male mice responded differently. Female mice decreased in delta body weight in response to the HFD, whereas male mice gained weight in response to the dietary challenge. Conversely, line CC005 exhibited the opposite response; female mice increased in weight, whereas male mice exhibited a decrease in weight in response to maintenance on the HFD. In lines CC010 and CC037 an increase in weight was observed among both the sexes of these lines maintained on a HFD. Line CC019 and the CC mean revealed a similar response of female mice increasing in weight in response to the HFD, whereas males appeared unaffected by the HFD compared to CHD counterparts. Thus, these results strengthen the genetic impact on weight gain and obesity development, and sex is also a substantial factor. Similar results were observed by Ghnaim et al.^38^

Consistent with previous studies, we observed a wide range of responses in body weight and glucose tolerance among different CC mouse lines in response to a HFD. Some lines exhibited a significant increase in body weight and glucose intolerance, whereas others exhibited minimal changes or even improvements in these parameters. These findings suggest that genetic factors play a critical role in determining susceptibility to obesity and T2D in response to dietary challenges. As a result of the widespread obesity pandemic, numerous comorbidities that threaten public health and impede the health‐care system have increased. Of the many conditions associated with obesity, T2D is of great interest as it is regarded as a global epidemic. T2D is a complex disease as several factors, such as genetic composition, epigenetics, and environment, contribute to its prevalence, and each element is yet to be fully understood. This research assessed glucose tolerance at two distinct time points, weeks 6 and 12. Variation in response to line and sex effect is present. This study was the first to demonstrate that the overall CC mean exhibited a significant increase in the AUC values in mice maintained on a HFD compared to CHD‐fed mice. When the line effect was considered, an increase in HFD‐maintained mice revealed higher AUC values than mice maintained on a CHD. All lines in this case exhibited an increased variation level, thus attributing to the difference in genetic composition. Previous research conducted on the C57BL/6J mouse model showed similar results.^46^

The relationships between organ weight, body weight, and diabetes have been reported previously,^38^ but the genetic background was not considered. The focus of this study was on how genetics affects organ weight in obesity and diabetes, which are brought on by a strict diet and exercise. Our results also revealed variations in organ weight, including the brain, liver, spleen, and kidneys, among different CC mouse lines in response to a HFD. Interestingly, certain lines exhibited alterations in organ weight that were consistent with metabolic dysfunction, such as increased liver weight, whereas others exhibited no significant changes. These findings further emphasize the influence of genetic background on metabolic homeostasis and organ function.

Our research found that the various tested lines reacted differently to glucose tolerance tests. Previous published work^24^ confirms these findings. Because genetic backgrounds differ and fluctuate, there is heterogeneity in how different people react to glucose tolerance testing. The HFD intake caused a significant decrease in glucose clearance in both populations, with the decline in the male population being more pronounced; other research studies have shown this to be the case.

Mice maintained on a HFD developed obesity, hyperinsulinemia, hyperglycemia, and hypertension. Although the control groups were fed ad libitum a standard CHD, metabolic abnormalities did not develop.^47^ The CC mean remained higher among mice maintained on a HFD than CHD counterparts at the second time point. Lines C57BL/6J, CC037, and CC010 revealed the same phenotype at time point 6; however, some alterations are present among the lines assessed. Line CC005 exhibited a decrease in AUC value in response to a HFD at time point 12, even though at time point 6, an increase was observed. These findings further confirm our prior research, highlighting the significance of the host genetic background and its substantial influence on mouse susceptibility to IR and increased body weight.^48^

The pancreas is the primary organ involved in T2D development. However, metabolic abnormalities are also observed in these organs; other organs such as the liver, skeletal muscles, kidney, brain, small intestine, and adipose tissue also play a role.^48^ Thus, changes in the weight of the brain, liver, kidney, and spleen were evaluated to gain a better understanding of the effects that obesity and excessive glucose levels have on the body and the development of MI. This study shows that distinct CC lines exhibit diverse reactions to the dietary challenge, emphasizing the role of genetic makeup in developing these diseases, also revealed earlier.^49^ Furthermore, the sex effect is evident because male and female mice of the same line exhibit different reactions.

In addition to metabolic phenotypes, our study investigated the impact of a HFD on cognitive function using the MWM test. We observed line‐specific differences in spatial learning and memory performance, indicating that genetic factors modulate susceptibility to diet‐induced cognitive deficits. Furthermore, given the well‐established link between metabolic dysfunction and AD, our findings suggest that genetic background may also influence the progression from metabolic disorders to neurodegenerative diseases such as AD. A growing body of research has revealed a link between obesity, T2D, and cognitive impairments, such as AD.^14^, ^45^ Individuals diagnosed with T2D face an elevated risk of developing AD, primarily due to shared glucose metabolic abnormalities.^50^ Previous studies have identified an increased risk of developing dementia in correlation with a T2D diagnosis. Furthermore, T2D has been linked to the degradation of brain gray matter, elevating the risk of developing many brain diseases. Degradation of the hippocampus, an integral region in memory function, can lead to MI that may develop into AD later on.^14^ The early stages of neurodegenerative diseases, like AD, typically present as memory loss that progressively evolves into broader cognitive and attention impairments over time.^14^ In addition to brain structure changes, specific organs indicate T2D and potentially AD development. To assess MIs, Y maze and MWM tests were conducted to evaluate the impact the HFD challenge has on the ability to perform these tasks. In most cases, the HFD improved overall performance. Additionally, correlation matrices revealed noteworthy differences in response to the dietary challenge.

The findings of our study have important implications for precision medicine and the development of targeted therapeutic interventions for obesity, T2D, and cognitive disorders. By elucidating the genetic factors that underlie individual variability in disease susceptibility and progression, our results provide valuable insights into potential therapeutic targets for intervention. Furthermore, our study highlights the importance of considering genetic background in preclinical research and drug development to improve the efficacy and safety of therapeutic interventions.

In conclusion, our study provides novel insights into the role of genetic background in mediating the phenotypic response to diet‐induced metabolic and cognitive disorders. The findings presented here highlight the importance of genetic diversity in shaping individual susceptibility to disease and underscore the need for personalized approaches to disease prevention and treatment. By elucidating the complex interplay between genetic and environmental factors, our study contributes to our understanding of the pathogenesis of obesity, T2D, cognitive deficits, and AD, and holds promise for the development of targeted therapeutic interventions.

## AUTHOR CONTRIBUTIONS

**Avia Paz:** Investigation; writing – original draft. **Kareem Midlej:** Data curation; formal analysis. **Osayd Zohud:** Formal analysis; investigation. **Iqbal M. Lone:** Data curation; methodology; validation; writing – original draft; writing – review and editing. **Fuad A. Iraqi:** Conceptualization; data curation; funding acquisition; investigation; methodology; project administration; resources; supervision; validation; writing – original draft; writing – review and editing.

## FUNDING INFORMATION

This work was supported by core funding from Tel‐Aviv University (TAU).

## CONFLICT OF INTEREST STATEMENT

The authors declare no conflict of interest.

## ETHICS APPROVAL AND CONSENT TO PARTICIPATE

The Institutional Animal Care approved all experiments in this study and Use Committee (IACUC) at TAU, which adheres to the Israeli guidelines and follows the NIH/USA animal care and use protocols (approved experiment number: TAU‐MD‐IL‐2205‐160‐5).

## CONSENT FOR PUBLICATION

Not applicable.

## Supporting information


Data S1.
