# Supplementary material for: The collaborative cross mouse for studying the effect of host genetic background on memory impairments due to obesity and diabetes
Source: Animal Model Exp Med. 2024 Oct 28;8(1):126–41. doi: 10.1002/ame2.12488 (PMC11798739; doi:10.1002/ame2.12488)

**Supplementary Figure 1.** The Y maze comprises three arms: a novel, a familiar, and the start arm. Mice are placed in the start arm when the novel arm is closed off, known as learning. During the test, all arms are open to allow the mouse to explore the arena freely. The novel arm and familiar arm are alternated between subjects to decrease bias to a particular side, as represented by the arrow.

**
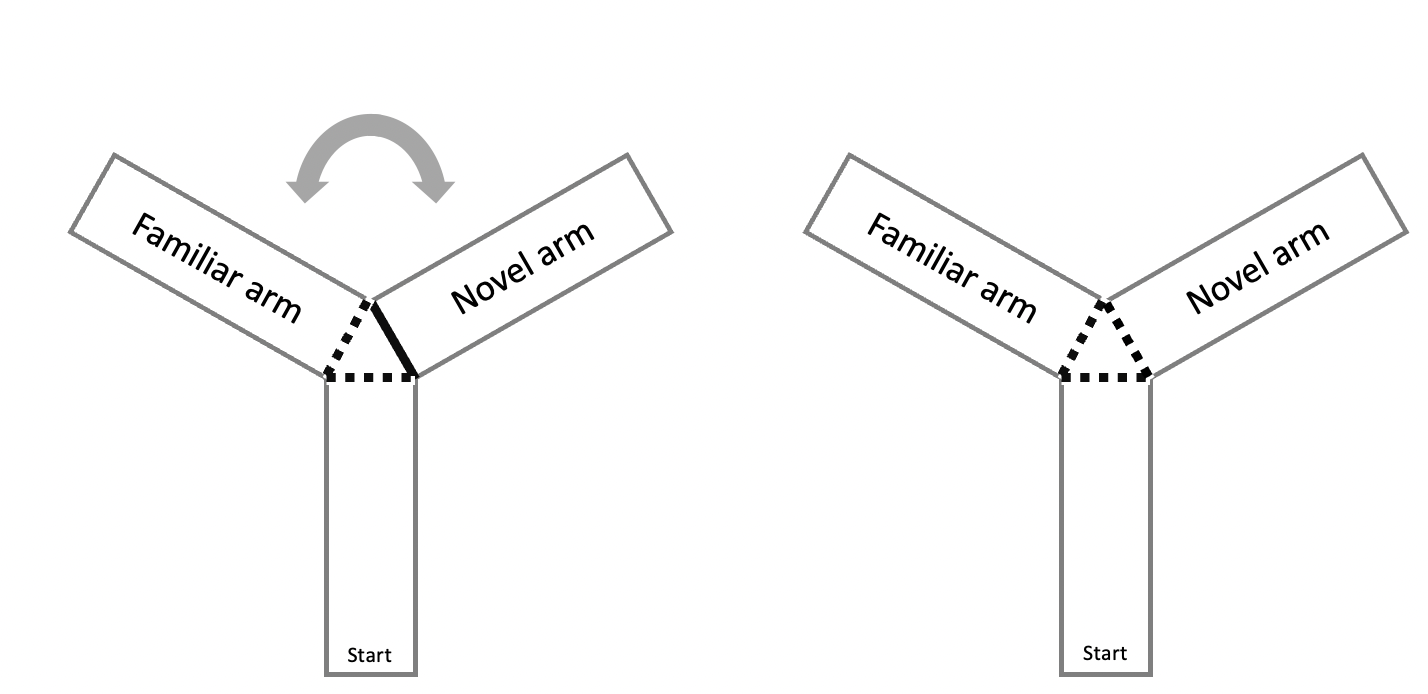
**

**Supplementary Figure 2.** The MWM consists of 5 days of learning in which each mouse is placed in a quadrant given one minute to locate the platform within 60 seconds, if the mouse does not locate the platform, the experimenter must guide the mouse to the platform, and wait 20 seconds before inserting to the next quadrant. Each day, each mouse is placed three times in the maze in 3 different quadrants for 3 minutes, each run spanning a minute. On day 6 the platform is removed. Mice are placed in the region opposite the platform region once for one minute. The software calculates the areas in which they are found. Regions in grey display the three quadrants the mouse is placed in on that day of the experiment (counterclockwise). On day one, the mice are placed first in quadrant I, II, and then III on day 2, the mouse is placed in II, III, IV, and so on.

Supplementary Figure 3**. Alterations in Percent Delta Body Weight Response to the HFD Challenge among the CC lines and C57BL/6J controls and Sex Effect. Figure 3A** shows the body weight changes (g) following 12 weeks of diet challenge CHD Vs. HFD of 4 different CC lines and C57BL/6J, as shown on the X-axis. The Y-axis represents ∆BW(g) calculated as $\frac{BW12(g)- BW0 (g).}{BW0}*100.$ Exact P values indicated if found significant. **Figure 3B** shows the body weight changes (g) following 12 weeks of diet challenge CHD Vs. HFD of the CC mean The Y-axis represents ∆BW(g) calculated as the $\frac{BW12(g)- BW0 (g).}{BW0}*100.$ Exact P values indicated if found significant as well as alternative results among lines. CC010 female and male mice of this line increased in percent delta body weight in response to an HFD.

**
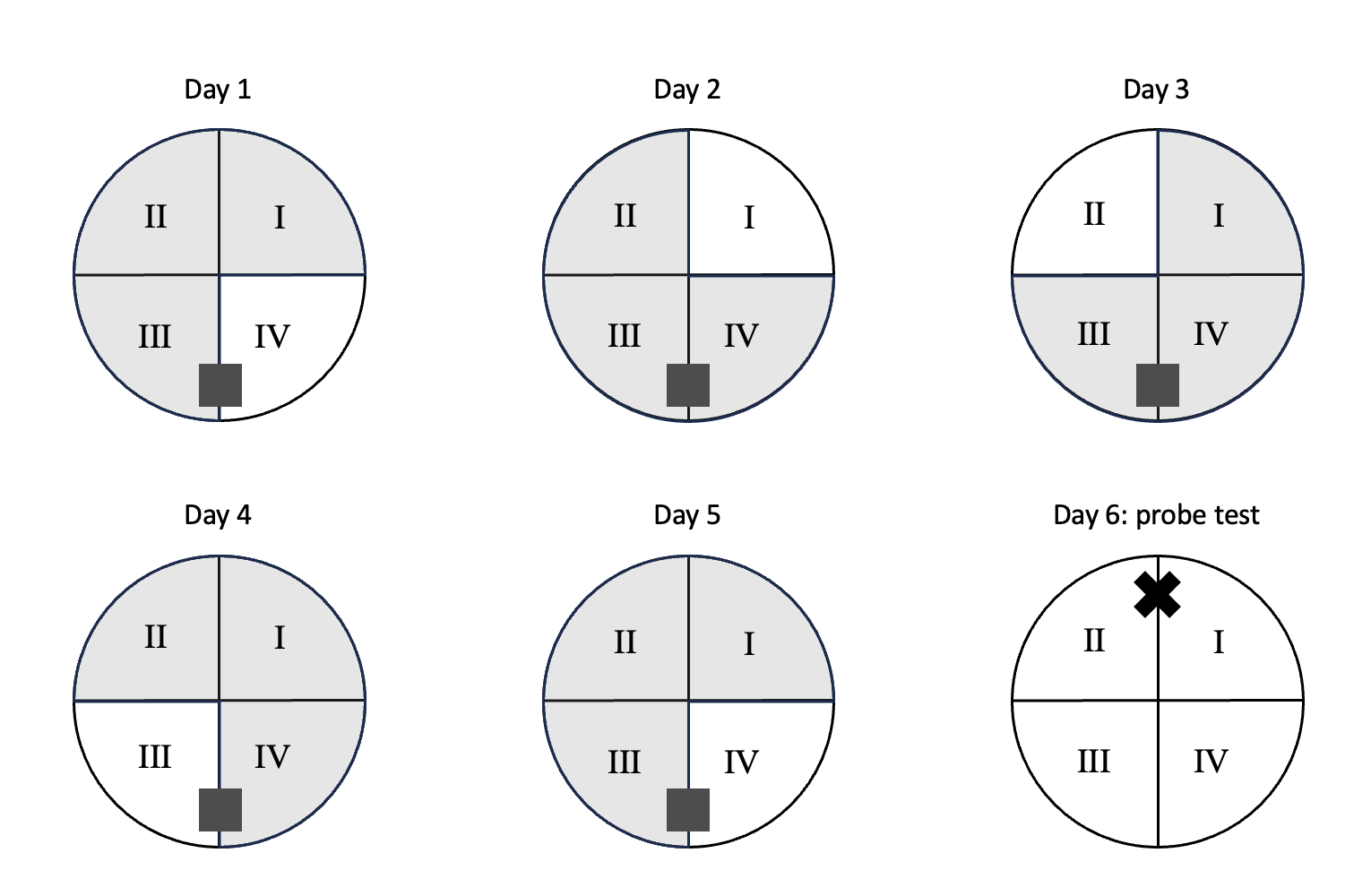
**

**Supplementary Figure 3.** Alterations in Percent Delta Body Weight Response to the HFD Challenge among the CC lines and C57BL/6J controls and Sex Effect. Figure 3A shows the body weight changes (g) following 12 weeks of diet challenge CHD Vs. HFD of 4 different CC lines and C57BL/6J, as shown on the X-axis. The Y-axis represents ∆BW(g) calculated as $\frac{BW12(g)- BW0 (g).}{BW0}*100.$ Exact P values indicated if found significant. **Figure 3B** shows the body weight changes (g) following 12 weeks of diet challenge CHD Vs. HFD of the CC mean The Y-axis represents ∆BW(g) calculated as the $\frac{BW12(g)- BW0 (g).}{BW0}*100.$ Exact P values indicated if found significant as well as alternative results among lines. CC010 female and male mice of this line increased in percent delta body weight in response to a HFD.


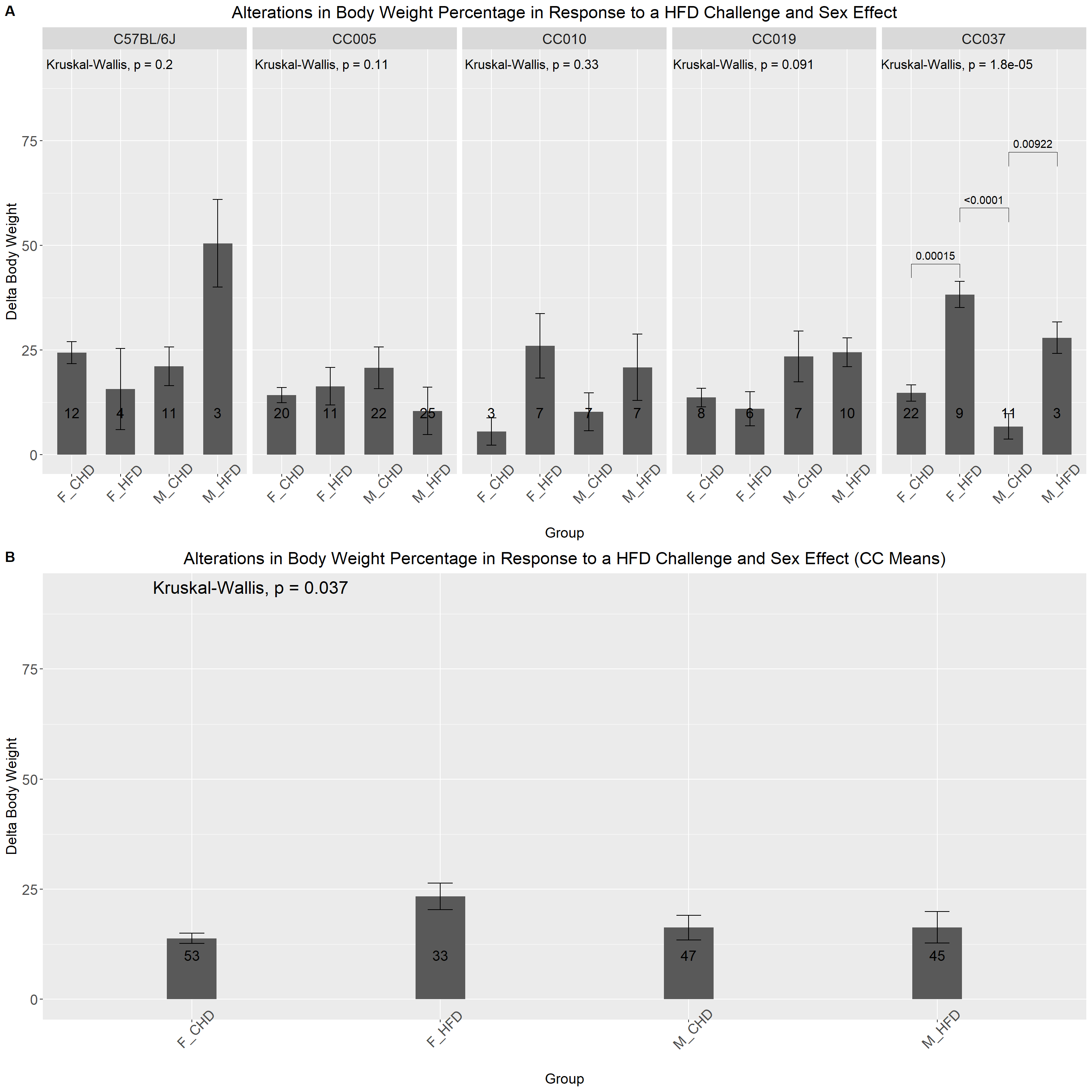


**Supplementary Figure 4.** Influence of the Dietary Challenge on Glucose Tolerance at 6 Weeks among the CC lines and C57BL/6J controls and Sex Effect. Figure 4A. shows the total area under the curve (AUC_0−180_) of glucose clearance (min*mg/dL) of intraperitoneal glucose tolerance test (IPGTT) at week 6 of four different CC lines divided by sex (male/female) after maintenance either on HFD (42 % Fat) challenge or CHD (18% fat). The X-axis presents CC lines and C57BL/ 6 controls, while the Y-axis presents AUC. Significant P values indicated. **Figure 4B.** shows the total area under the curve (AUC_0−180_) of glucose clearance (min*mg/dL) of intraperitoneal glucose tolerance test (IPGTT) at week 6 of the CC divided by sex (male/female) after maintenance either on HFD (42 % Fat) challenge or CHD (18% fat). The X-axis presents CC lines and the mean; the Y-axis presents AUC. Significant P values indicated.


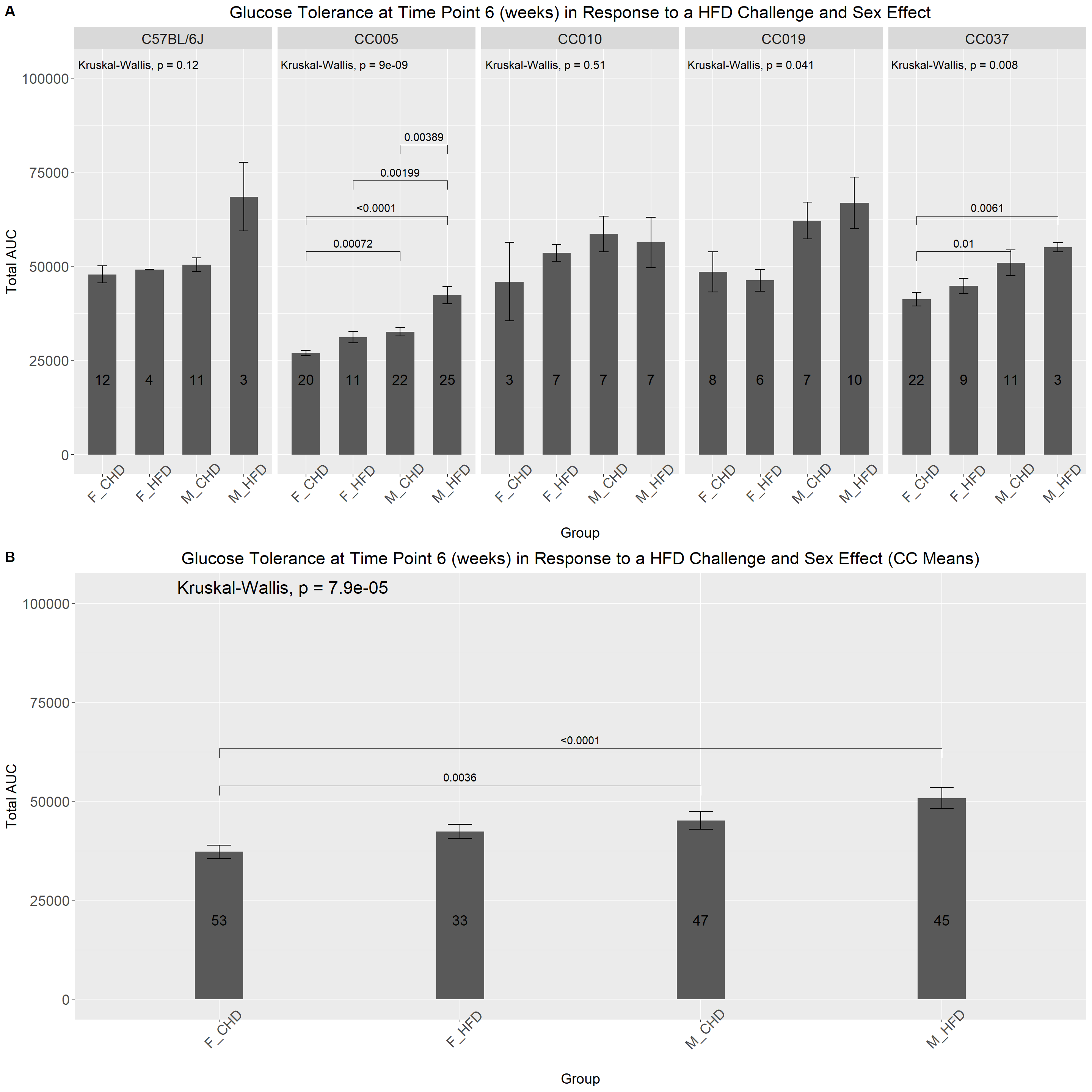


**Supplementary Figure 5.** Influence of the Dietary Challenge on Glucose Tolerance at 12 Weeks among the CC lines and C57BL/6J controls and Sex Effect. Figure 5A shows the total area under the curve (AUC_0−180_) of glucose clearance (min*mg/dL) of intraperitoneal glucose tolerance test (IPGTT) at week 12 of four different CC lines and C57BL/ 6 divided by sex (male/female) after maintenance either on HFD (42 % Fat) challenge or CHD (18% fat). The X-axis presents CC lines and C57BL/ 6 controls; the Y-axis presents AUC. Significant P values indicated. Kruskal-Wallis + dunn_test multiple comparisons were conducted**. Figure 5B** shows the total area under the curve (AUC_0−180_) of glucose clearance (min*mg/dL) of intraperitoneal glucose tolerance test (IPGTT) at week 12 of the CC mean divided by sex (male/female) after maintenance either on HFD (42 % Fat) challenge or CHD (18% fat). The X-axis presents CC lines and the mean; the Y-axis presents AUC. Significant P values indicated. Kruskal-Wallis + dunn_test multiple comparisons were conducted.


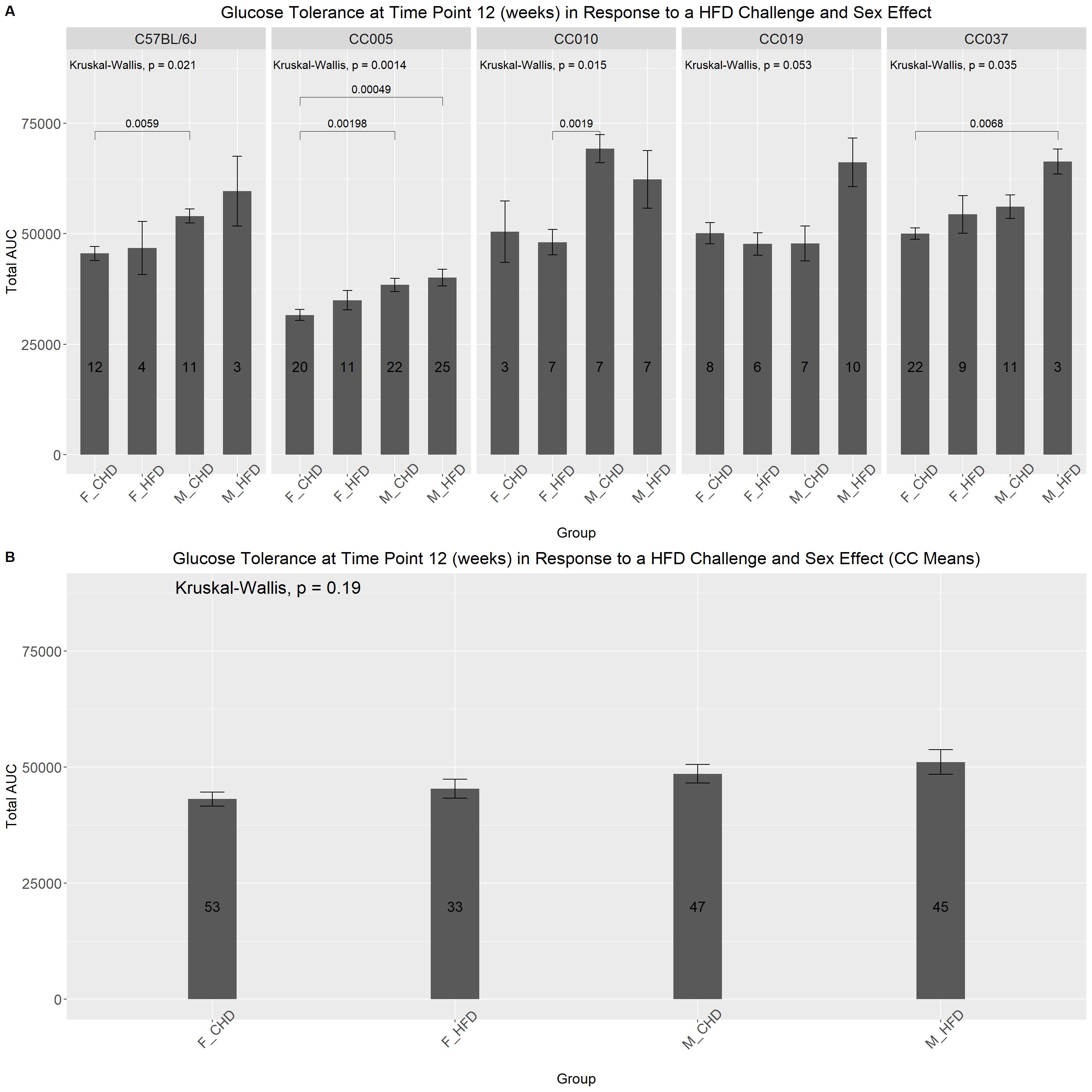


**Supplementary Figure 6.** Influence of the Dietary Challenge on Adjusted Brain Weight among the CC lines and C57BL/6J controls and Sex Effect. Figure 4A**.** shows the brain weight measured at the termination of the experiment and adjusted to the final body weight (Adj. Brain weight) of CC line mice and C57BL/ 6 after maintenance either on HFD (42 % Fat) challenge or CHD (18% Fat). The X-axis presents CC lines and C57BL/ 6 controls divided by sex (female/male); the Y-axis presents adj brain weight. Significant P values indicated. Kruskal-Wallis + dunn_test multiple comparisons were conducted. **Figure 6B.** shows the brain weight measured at the termination of the experiment and adjusted to the final body weight (Adj. Brain weight) of the CC mean after maintenance either on the HFD (42 % Fat) challenge or CHD (18% Fat). The X-axis presents CC mean divided by sex (female/male); the Y-axis presents adj brain weight. Significant P values indicated. Kruskal-Wallis + dunn_test multiple comparisons were conducted.


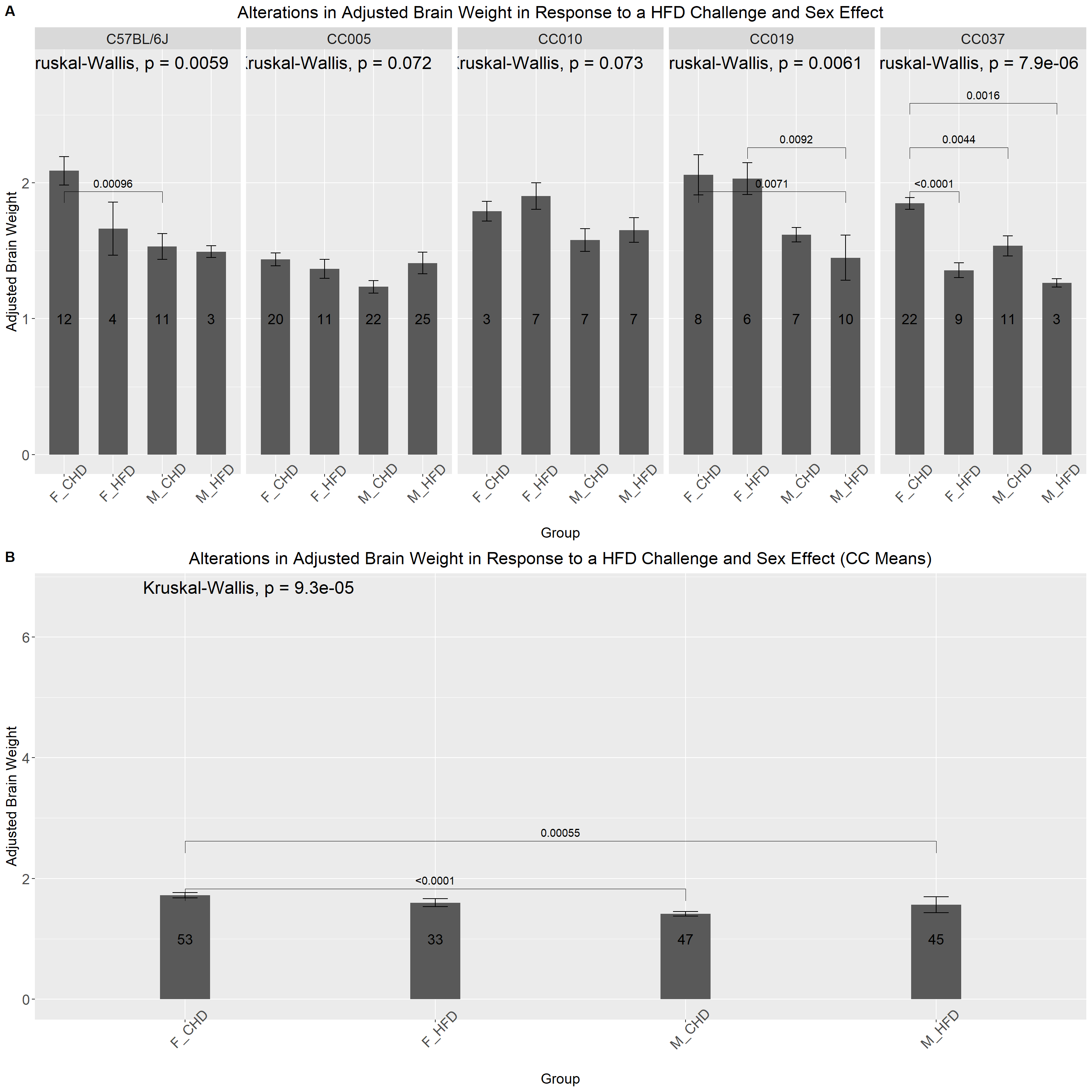


**Supplementary Figure 7.** Influence of the Dietary Challenge on adjusted spleen weight among the CC lines and C57BL/6J controls and Sex Effect. Figure 7A shows the spleen weight measured at termination of the experiment and adjusted to final body weight (Adj. Brain weight) of CC line mice and C57BL/ 6 after maintenance either on HFD (42 % Fat) challenge or CHD (18% fat). The X-axis presents CC lines and C57BL/ 6 controls divided by sex (female/male); the Y-axis presents adj brain weight. Significant P values indicated. Kruskal-Wallis + dunn_test multiple comparisons were conducted. **Figure 7B** shows the spleen weight measured at termination of the experiment and adjusted to final body weight (Adj. Brain weight) of the CC mean after maintenance either on HFD (42 % Fat) challenge or CHD (18% fat). The X-axis presents CC mean divided by sex (female/male); the Y-axis presents adj brain weight. Significant P values indicated. Kruskal-Wallis + dunn_test multiple comparisons were conducted.


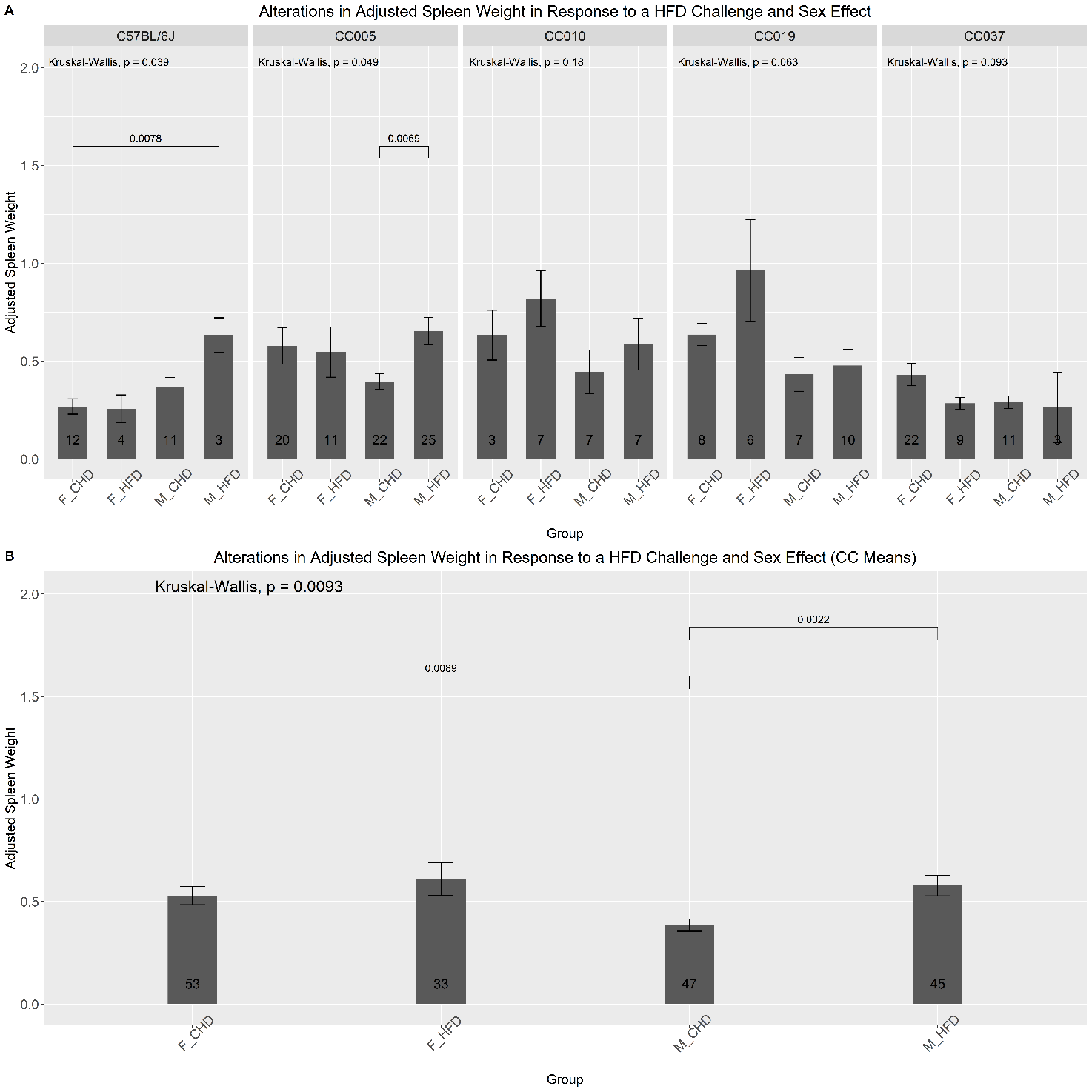


**Supplementary Figure 8.** Influence of the Dietary Challenge on adjusted Liver weight among the CC lines and C57BL/6J controls and Sex Effect. Figure 8A. shows the liver weight measured at the termination of the experiment and adjusted to final body weight (Adj. Brain weight) of CC line mice and C57BL/ 6 after maintenance either on HFD (42 % Fat) challenge or CHD (18% fat). The X-axis presents CC lines and C57BL/ 6 controls divided by sex (female/male); the Y-axis presents adj brain weight. Significant P values indicated. Kruskal-Wallis + dunn_test multiple comparisons were conducted. **Figure 8B.** shows the liver weight measured at termination of the experiment and adjusted to final body weight (Adj. Brain weight) of the CC mean after maintenance either on HFD (42 % Fat) challenge or CHD (18% fat). The X-axis presents CC mean divided by sex (female/male); the Y-axis presents adj brain weight. Significant P values indicated. Kruskal-Wallis + dunn_test multiple comparisons were conducted.

**
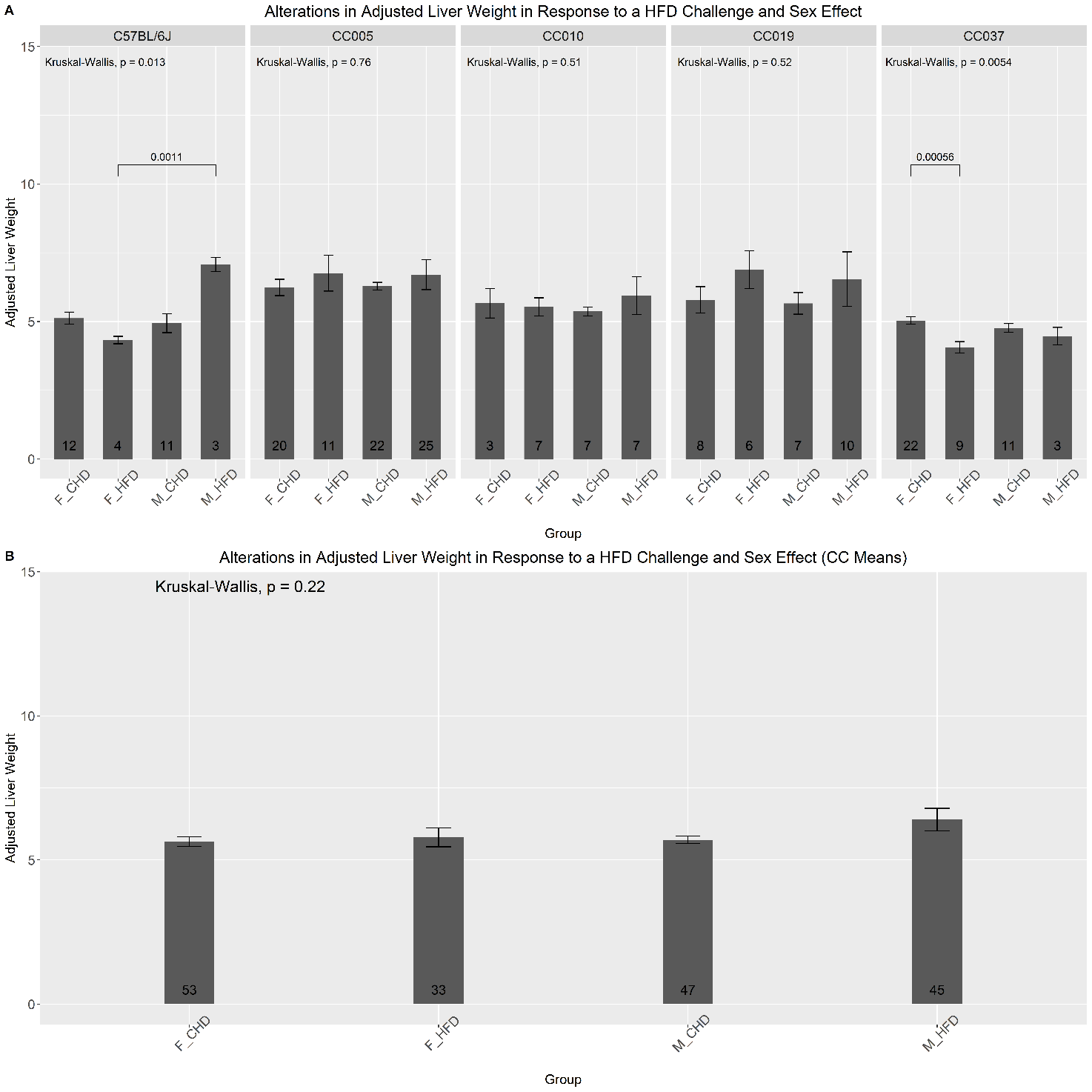
**

**Supplementary Figure 9.** Kidney: Influence of the Dietary Challenge on adjusted Kidney weight among the CC lines and C57BL/6J controls and Sex Effect. Figure 9A shows the kidney weight measured at termination of the experiment and adjusted to final body weight (Adj. Brain weight) of the CC lines and C57BL/ 6 after maintenance either on HFD (42 % Fat) challenge or CHD (18% fat). The X-axis presents CC lines and C57BL/ 6 controls divided by sex (female/male); the Y-axis presents adj kidney weight. Significant P values indicated. Kruskal-Wallis + dunn_test multiple comparisons were conducted. **Figure 9B** shows the kidney weight measured at termination of the experiment and adjusted to final body weight (Adj. Brain weight) of the CC mean after maintenance either on HFD (42 % Fat) challenge or CHD (18% fat). The X-axis presents CC mean divided by sex (female/male); the Y-axis presents adj kidney weight. Significant P values indicated. Kruskal-Wallis + dunn_test multiple comparisons were conducted.


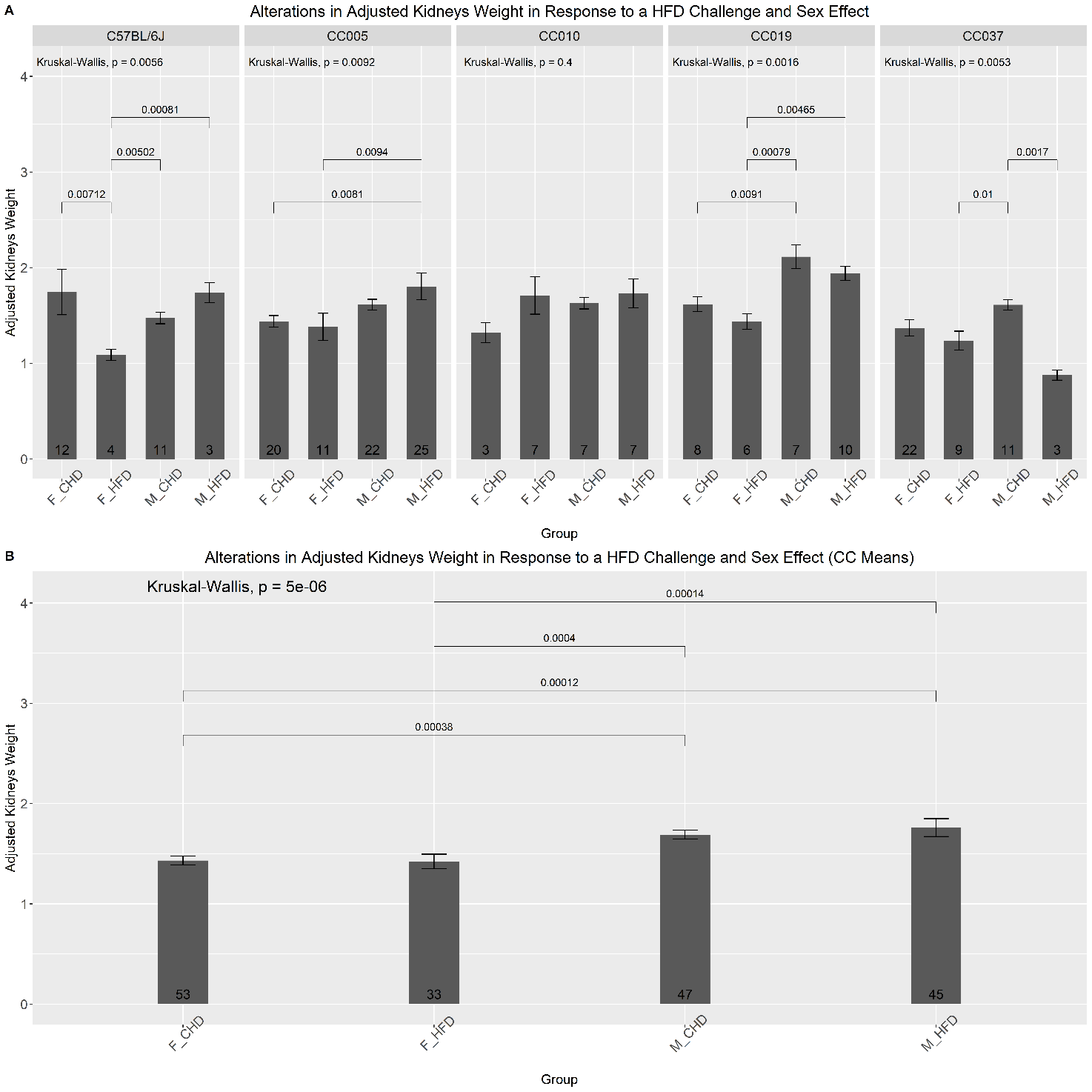


**Supplementary Figure 10.** This graph represents the preference index that ranges from -1 to 1. The closer the value to 1 the more time in the novel arm. The closer the preference index is to -1, the more time is spent in the familiar arm of the Y maze.Figure 10A shows the Preference index among the 5 different lines following maintenance either on HFD (42 % Fat) challenge or CHD (18% fat). The X-axis reveals the dietary challenge, and the Y-axis displays the index. Figure 10B shows the Preference index among the CC mean following maintenance either on HFD (42 % Fat) challenge or CHD (18% Fat). The X-axis reveals the dietary challenge, and the Y-axis displays the index.


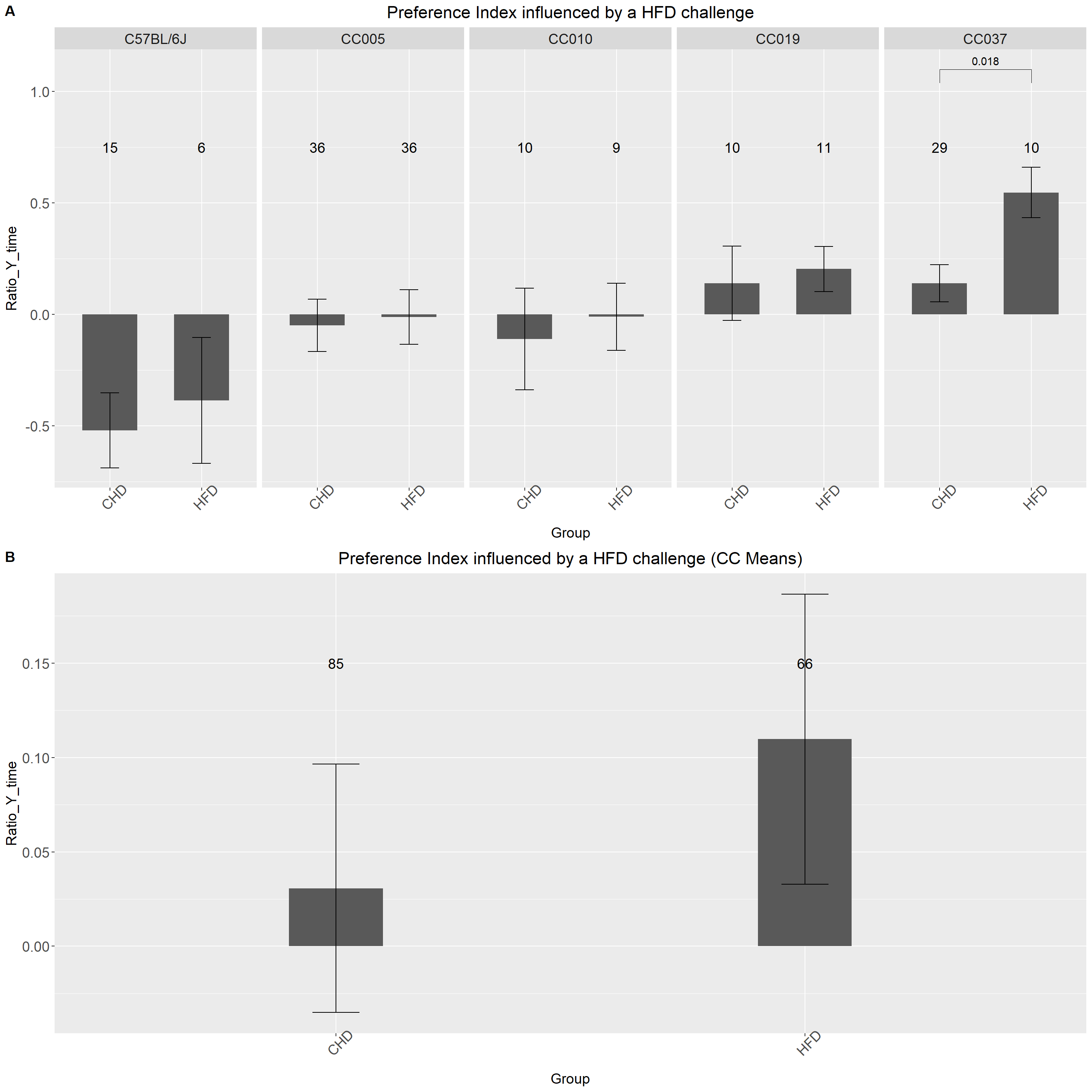


**Supplementary Figure 11: Preference Index in Response to an HFD and Sex Effect. Figure 11A shows** the Preference index among the 5 different lines following maintenance either on HFD (42 % Fat) challenge or CHD (18% fat). The X-axis reveals the dietary challenge and sex, and the Y-axis displays the index. **Figure 11B** shows the Preference index among the CC mean following maintenance either on the HFD (42 % Fat) challenge or CHD (18% Fat). The X-axis reveals the dietary challenge and sex, and the Y-axis displays the index.


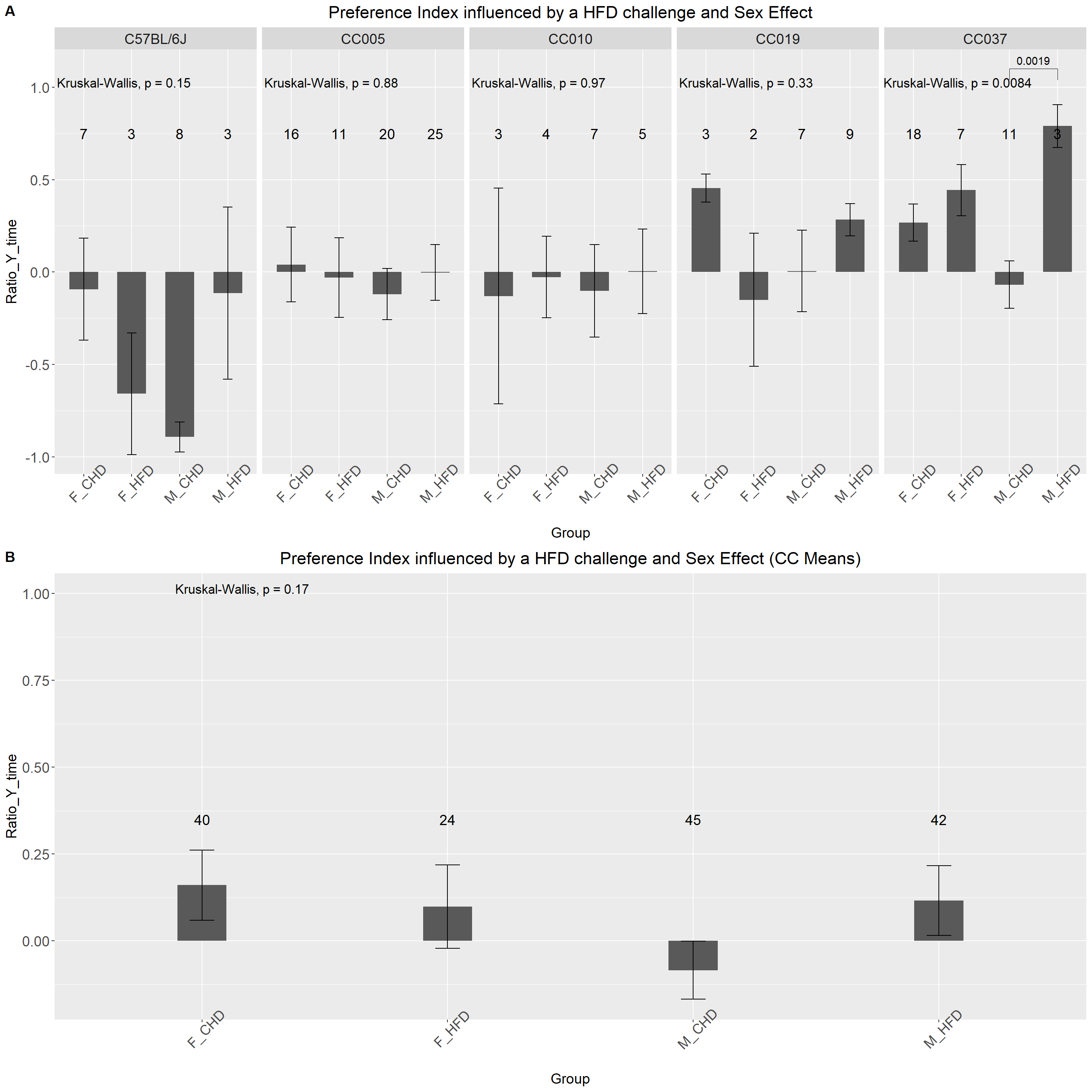


**Supplementary Figure 12: Morris Water Maze Days 1-6 of CC Line CC005.** The Following graphs represent the 6-day MWM of each line tested. Following maintenance either on HFD (42 % Fat) challenge or CHD (18% fat). Figure A represents the diet effect, and Figure B represents the sex and diet effect for each line. The X-axis represents the days (1-5) and the probe test on day 6; the Y-axis represents time in seconds. Significant P values indicated.

Shown in Figure **12**A. The dietary challenge appears not to influence the overall performance of line CC005, as the progress in learning the maze is similar. While in Figure **12**B**.** When sex and diet are considered, variation in response to the diet and between female and male mice is displayed. Yet, the overall progression is similar among male and female mice.


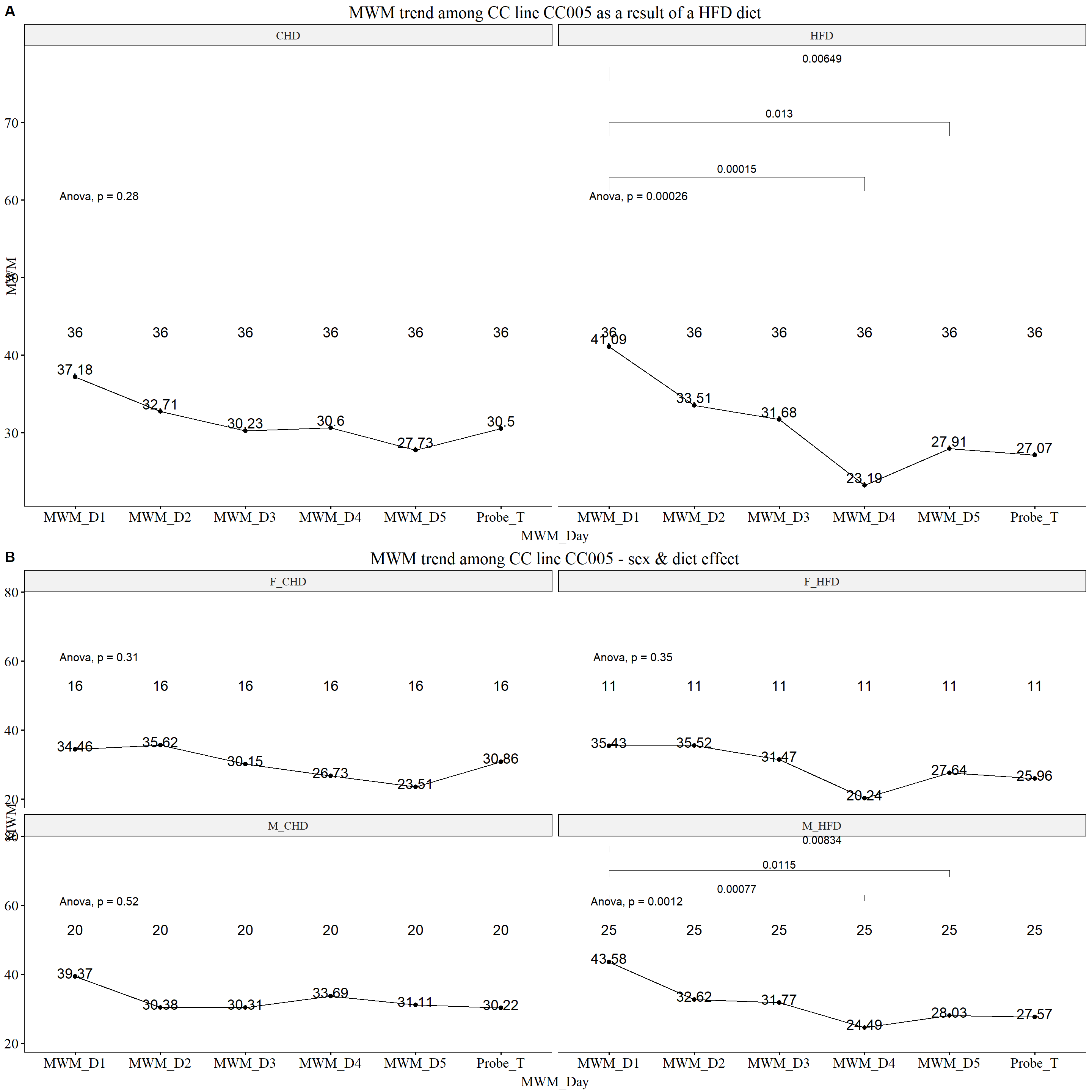


Supplementary Figure 1**3: Morris Water Maze Days 1-6 of CC Line CC019.** Shown in Figure **13**A. The dietary challenge impacts the mice of line CC019 as the performance of the maze is different when comparing the diets. Mice maintained on a CHD reveal an inconsistent learning curve and a regression in performance. Mice maintained on an HFD reveal an inconsistent learning curve; however, it improved over the course of the days. overall, the performance is better on an HFD, as shown in Figure **13**B. when sex and diet are considered, variation in response to the diet and between female and male mice is displayed. Among female mice, an improved performance is evident among those maintained on an HFD, while among males, the performance is also improved in response to the HFD; however, it is less extreme.


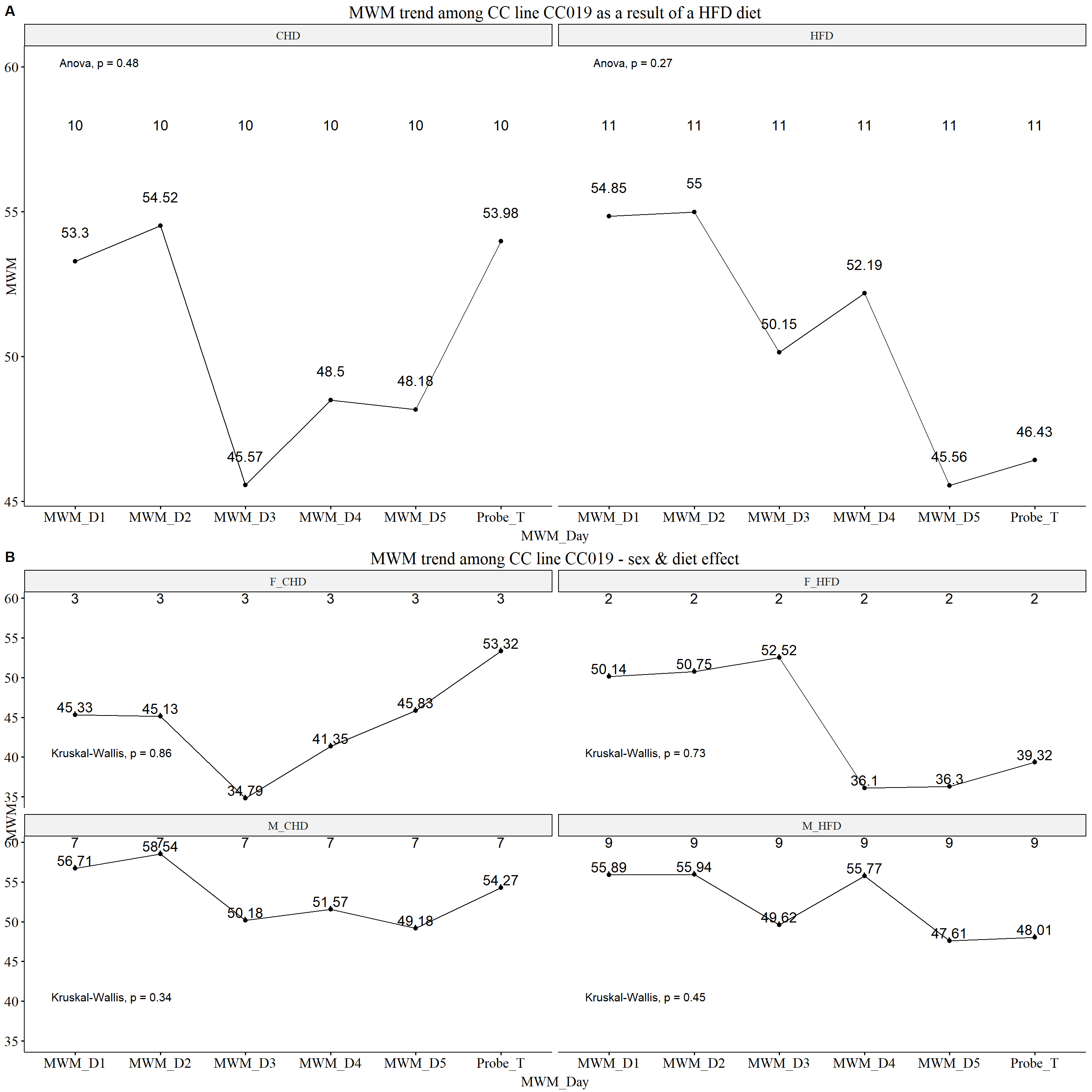


**Supplementary Figure 14: Morris Water Maze Days 1-6 of CC Line CC010.** In Figure 14A., the dietary challenge impacts the mice of line CC010 as the performance of the maze is different when comparing the diets. The learning curve among mice maintained on a CHD and a HFD could be more steady. Both reveal a regression in performance. Moreover, in figure **1**4B. when sex and diet are considered, variation in response to the diet and between female and male mice is displayed. The diet appears to negatively impact female performance on an HFD as a regression is evident. The HFD, however, improved the performance among male mice on an HFD when compared to CHD counterparts.


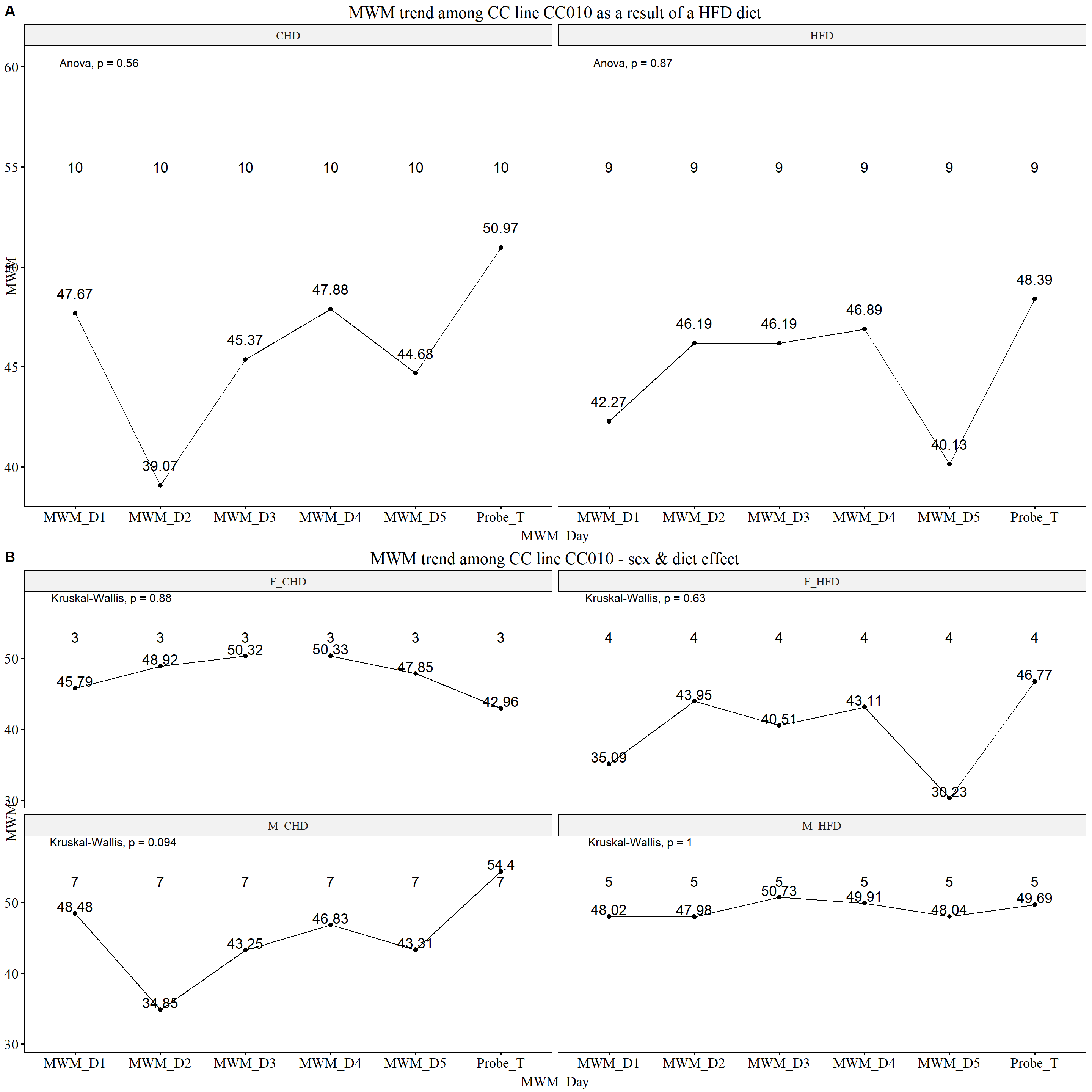


Supplementary Figure **15: Morris Water Maze Days 1-6 of C57BL/6J**. **15A shows that the dietary challenge impacts the mice of line C57BL/6J, as the performance of the maze is different when compared to** the diets. Mice maintained on a CHD reveal a steady learning curve, while HFD-maintained mice reveal a slight regression. Ultimately, this line performs the maze well. **15B shows that w**hen sex and diet are considered, variation in response to the diet and between female and male mice is displayed. However, all females and males of this line perform the maze well, and an improvement is seen as the days progress on both a CHD and an HFD.


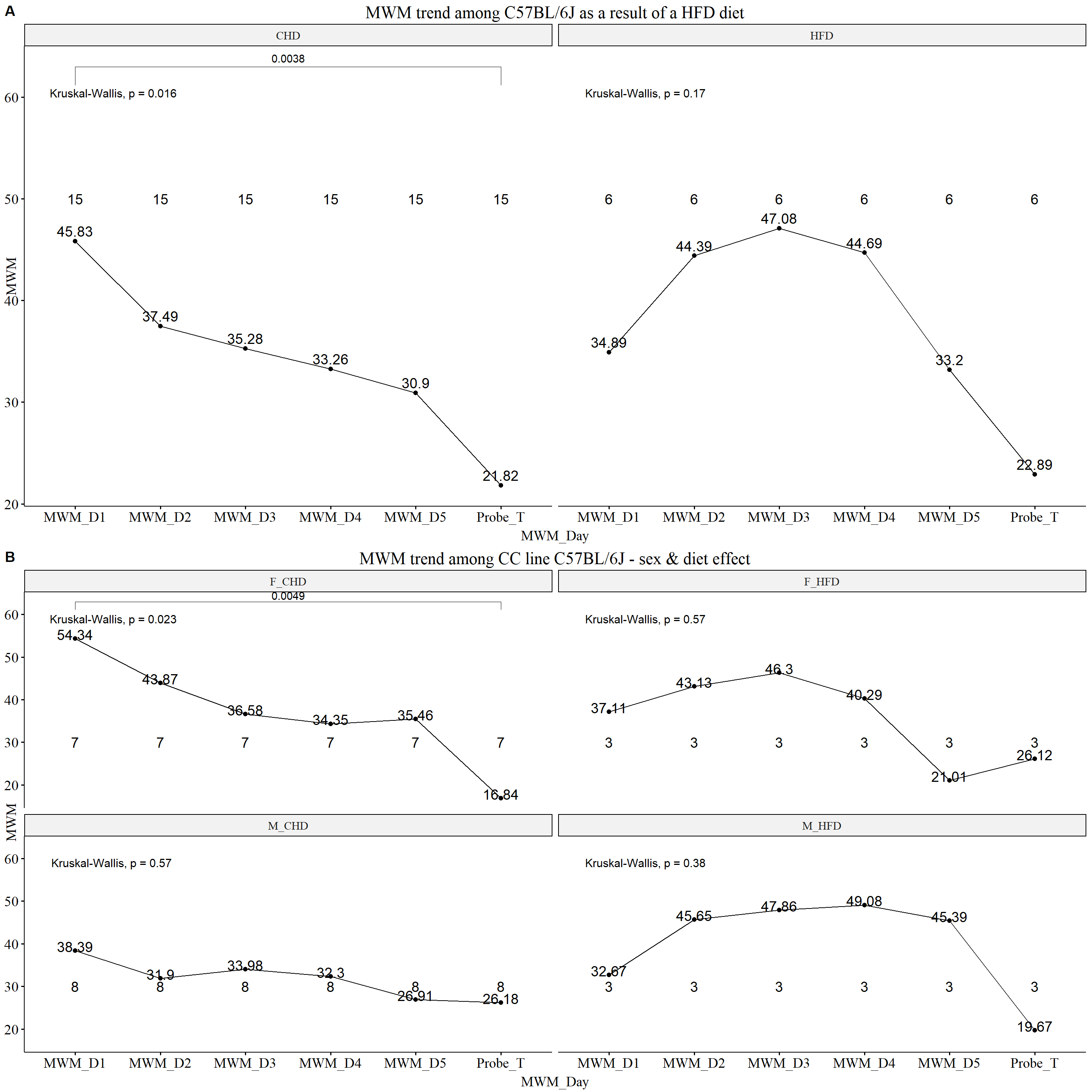


**Supplementary Figure 16:** Alterations in Latency to the probe region in response to an HFD. Figure 16A**.** shows the time to cross the platform region on the 6^th^ day of the experiment probe test of 4 CC lines and C57BL/ 6 controls after maintenance either on HFD (42 % Fat) challenge or CHD (18% Fat). The X-axis presents CC lines and C57BL/ 6 controls; the Y-axis represents the time to cross the platform region. Significant P values indicated. Wilcoxon test conducted. **Figure 16B.** shows the time to cross the platform region on the 6^th^ day of the experiment probe test of the CC mean after maintenance either on HFD (42 % Fat) challenge or CHD (18% Fat). The X-axis presents the CC mean; the Y-axis represents the time to cross the platform region. Significant P values indicated. Wilcoxon test comparisons were conducted.


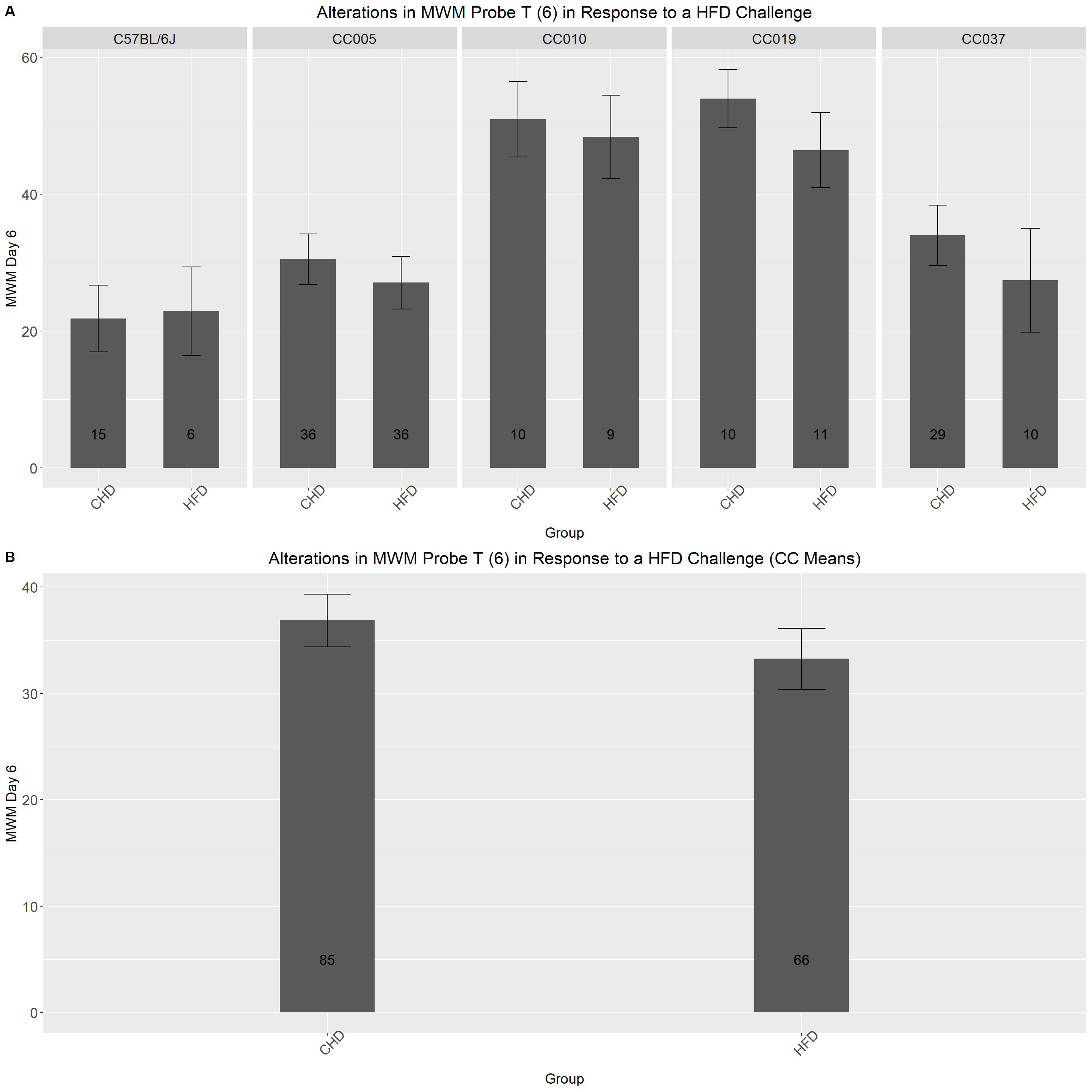


**Supplementary Figure 17:** Heatmap and Spearman correlation for CC mean of select traits of the CC population after maintained for 14 weeks either on HFD or CHD. Figure represents male mice maintained on HFD. The r values ranged between minimum (-1) and maximum (1).

**
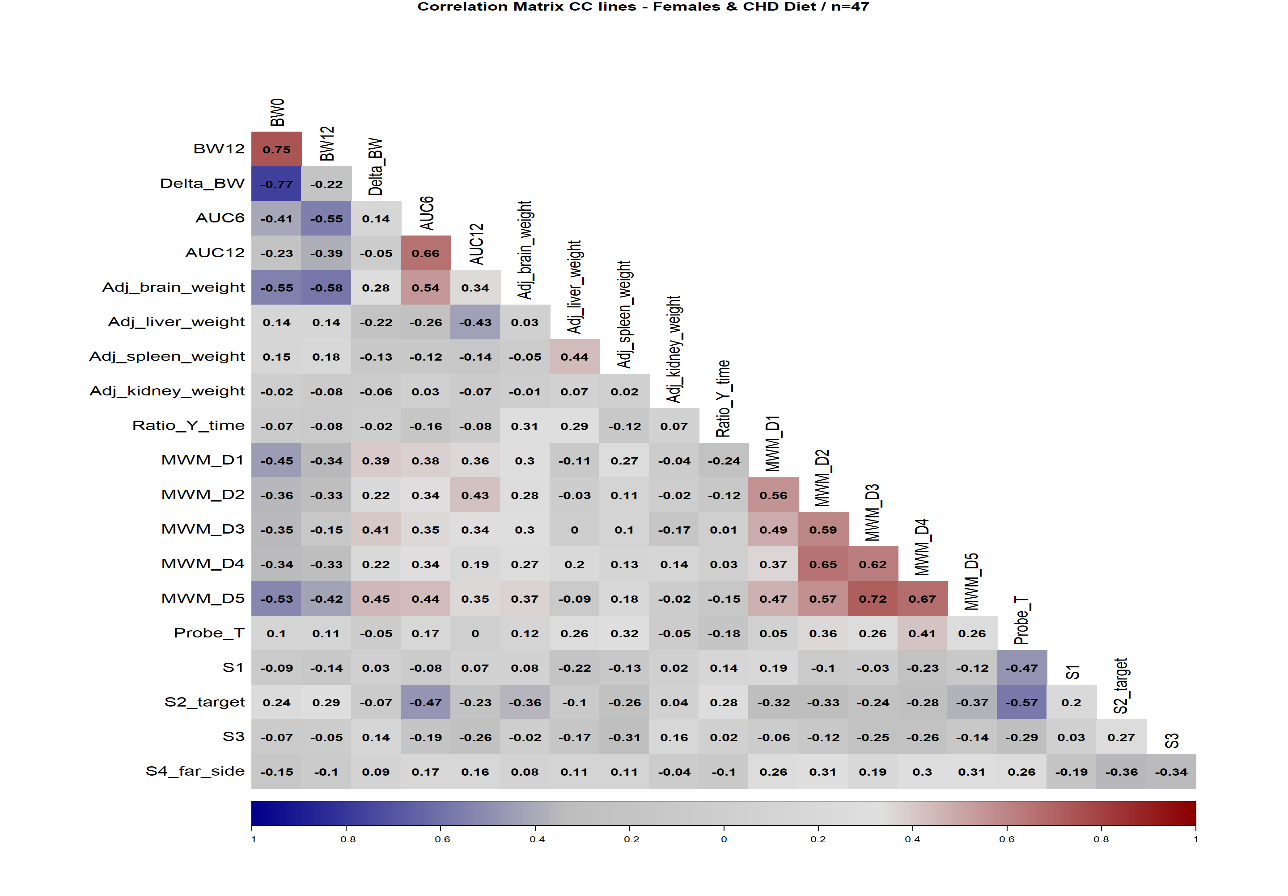
**

**Supplementary Figure 18:** Heatmap and Spearman correlation for CC mean of select traits of the CC population after maintained for 14 weeks either on HFD or CHD. Figure represents female mice maintained on CHD. The r values ranged between minimum (-1) and maximum (1).

**
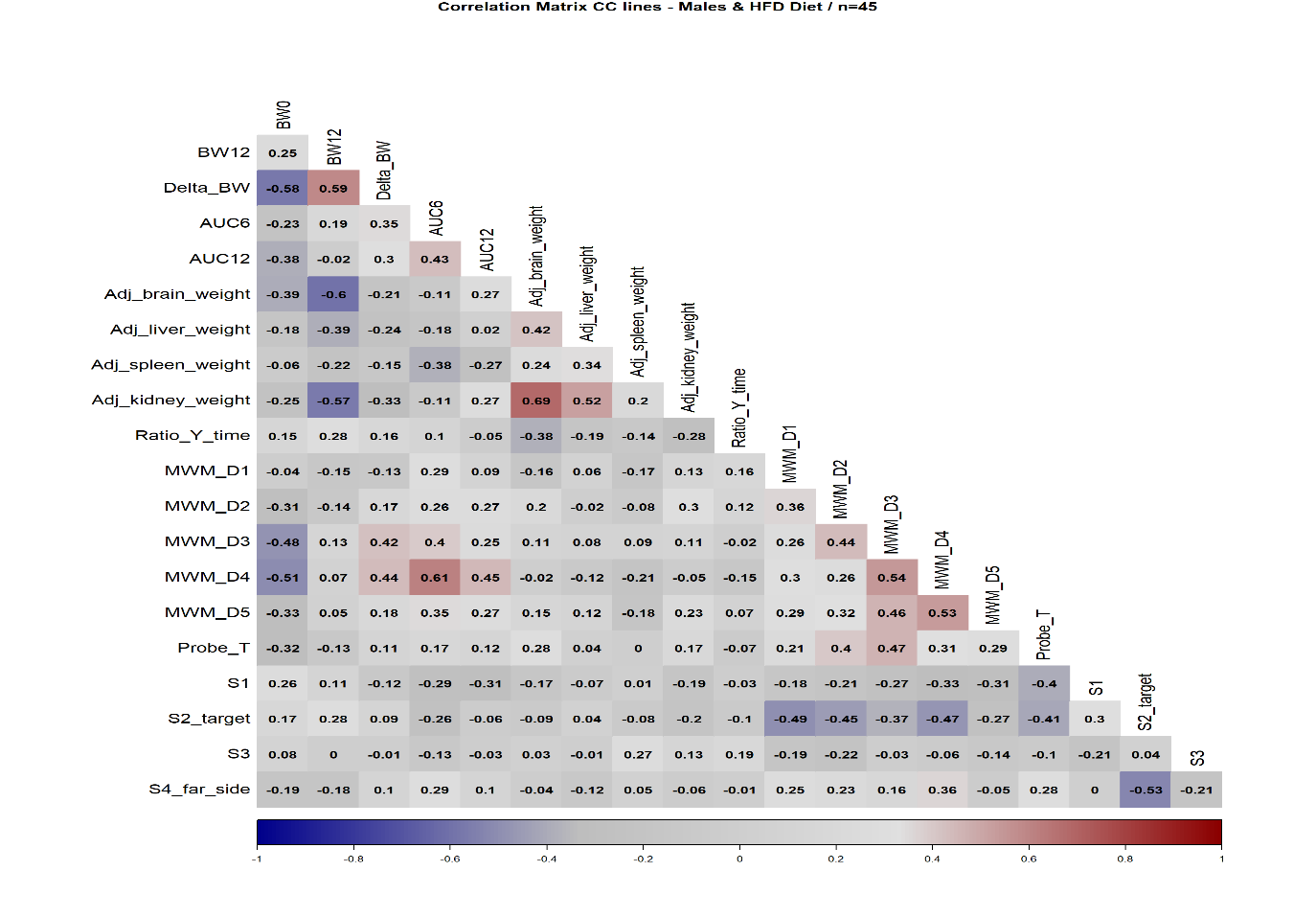
**

**Supplementary Figure 19:** Heatmap and Spearman correlation for CC mean of select traits of the CC population after being maintained for 14 weeks either on HFD or CHD. The figure represents female mice maintained on HFD. The r values ranged between minimum (-1) and maximum (1).


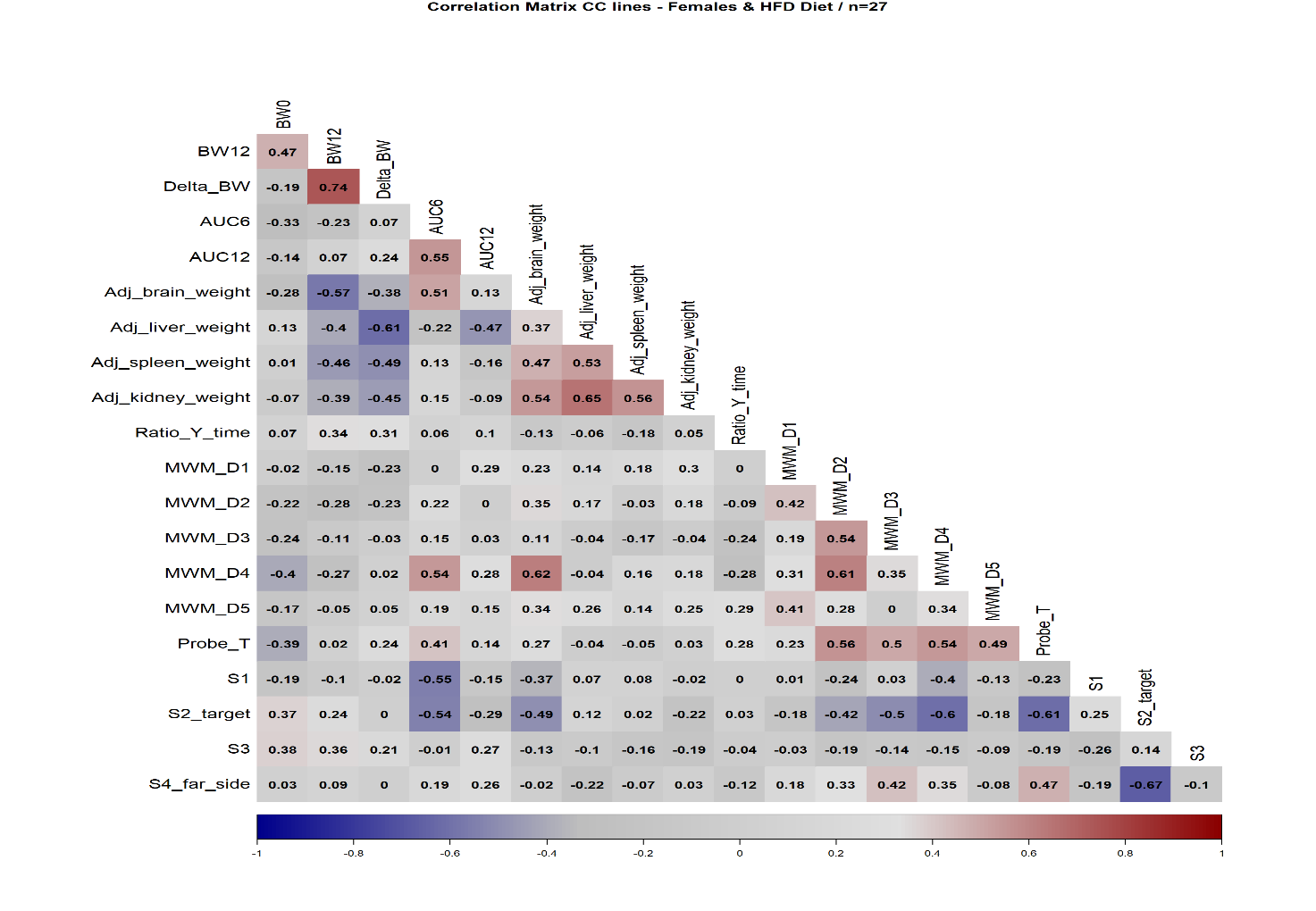


**Supplementary Figure 20:** Heatmap and Spearman correlation for select traits of the C57Bl/6J after maintained for 14 weeks either on HFD or CHD Figures (A) mice maintained on CHD; (B) mice maintained on HFD. The r values ranged between minimum (-1) and maximum (1).

A

B


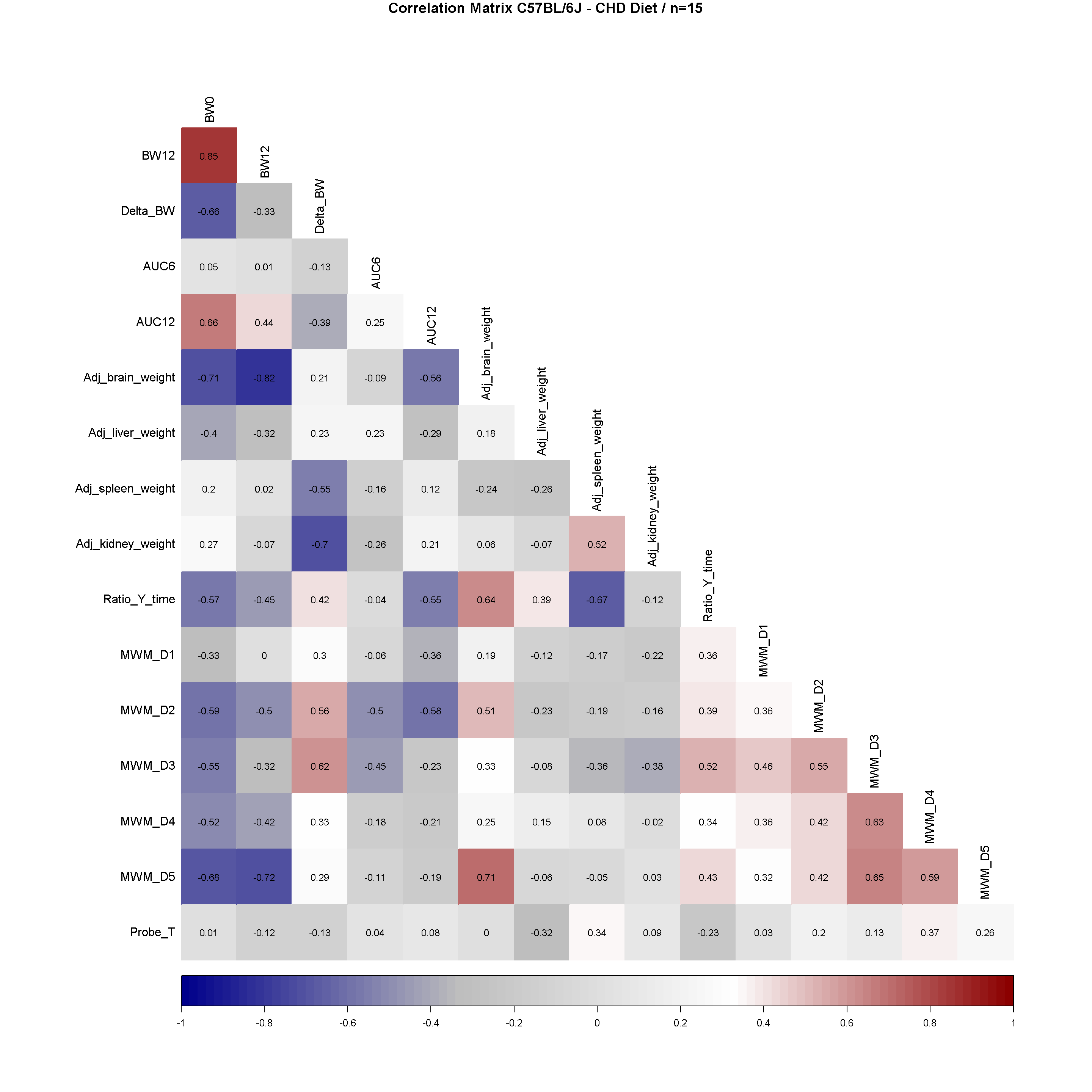

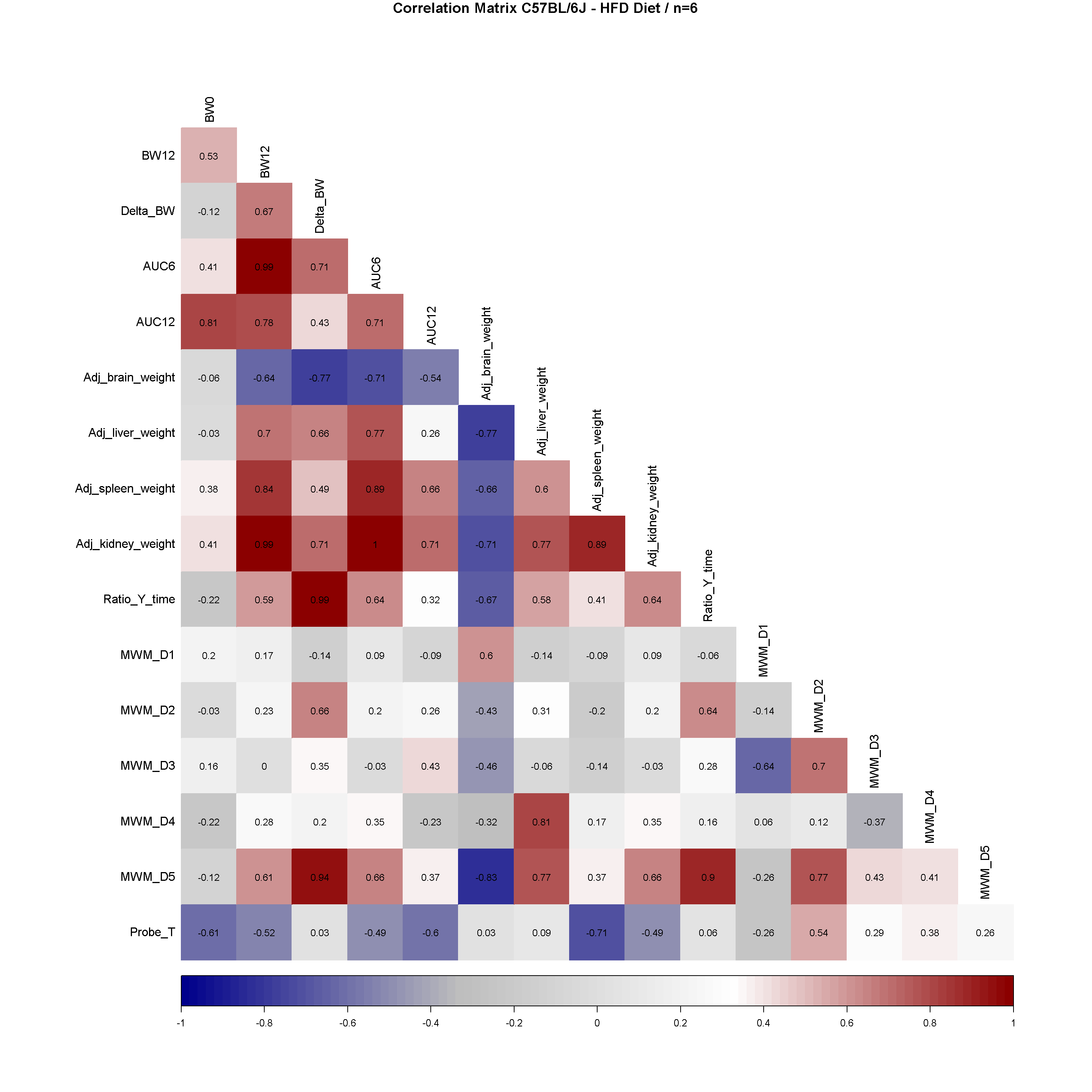

Supplement: Supplementary file 1 — Data S1. [file AME2-8-126-s001.docx]
